# Loss of *Fmr1* causes subnucleus-specific decreases in dendritic complexity in mediodorsal thalamus

**DOI:** 10.1007/s00429-026-03179-5

**Published:** 2026-10-14

**Authors:** Alena Kizimenko, Polina Lyuboslavsky, Audrey C. Brumback

**Affiliations:** 1 Department of Neurology, Dell Medical School at The University of Texas at Austin, Austin, TX USA; 2 Department of Pediatrics, Dell Medical School at The University of Texas at Austin, Austin, TX USA; 3 Center for Learning and Memory at The University of Texas at Austin, 1 University Station, Mail Code C7000, Austin, TX 78712 USA

**Keywords:** FMRP, fragile X syndrome, thalamocortical, Sholl analysis, prefrontal cortex, HCN channels

## Abstract

In the mediodorsal thalamus (MD), neurons of the medial (MD-M) and lateral (MD-L) subnuclei project to the medial prefrontal cortex (mPFC) to control complementary aspects of behavior, with MD-M supporting more of the social/emotional domain and MD-L promoting cognitive functions. Our recent work shows that MD-M and MD-L have differences in hyperpolarization-activated, cyclic nucleotide-gated (HCN) pacemaker channels that may enable their distinct roles in behavior. Low HCN channel activity in MD-M neurons makes them relatively sensitive and responsive to inputs, while higher HCN channel activity makes MD-L neurons more specific and selective for which inputs it transforms into outputs. Knockout (KO) of the fragile X syndrome-related gene *Fmr1* decreased the activity of HCN channels in MD-L but not MD-M neurons, making MD-L neurons more like MD-M cells physiologically. We sought to understand if these *Fmr1*-related changes in MD function were accompanied by changes in subcellular structure. Here, we assessed the dendritic morphology of MD neurons in adult *Fmr1* KO and wildtype (WT) littermate mice. In acute *ex vivo* brain slices, we filled MD neurons with biocytin via whole cell recording pipettes. We used Neurolucida to trace the dendrites, performed quantitative analysis of dendritic architecture, compared MD-M and MD-L neurons in wildtype mice, and compared WT to KO mice for each subnucleus. In WT mice, MD-M neurons showed more distal dendritic branching compared with MD-L neurons. In *Fmr1* KO mice, MD-M neurons lost their distal dendritic branching and exhibited significant reductions in multiple measures of dendritic complexity compared to WT controls (total dendritic length, number of nodes, and number of terminals), while MD-L neurons showed no morphological differences between WT and KO animals. Notably, these morphological changes show a double dissociation with our previous physiological findings: MD-M neurons exhibit structural but not functional changes, while MD-L neurons show functional but not structural changes in *Fmr1* KO mice. Together, these findings suggest that FMRP regulates structure and function through independent, circuit-specific mechanisms in thalamocortical neurons, with implications for understanding the diverse cellular and network consequences of *Fmr1* loss in fragile X syndrome.

**Supplementary Information:**

The online version contains supplementary material available at https://doi.org/10.1007/s00429-026-03179-5.

## Introduction

As part of the prefrontal network, the mediodorsal nucleus of the thalamus (MD) receives excitatory and inhibitory inputs from cortical and subcortical structures involved in cognitive and social/emotional functions (Grodd et al. 2020; Nelson et al. 2025). In rodents, MD subnuclei are named for their location and in primates for their cytoarchitecture (rodent/primate): medial (MD-M)/magnocellular (MDmc); central (MD-C)/parvocellular (MDpc); lateral (MD-L)/pars multiformis (MDmf) and densocellular (MDdc) (Krettek and Price 1977; Goldman-Rakic and Porrino 1985; Van 1986; Giguere and Goldman-Rakic 1988; Barbas et al. 1991; Mitchell and Chakraborty 2013; Onishi et al. 2022; Scott et al. 2024). In addition to these anatomical differences, data from across a range of experimental modalities suggest that MD subnuclei represent functionally distinct subdivisions, including differences in gene expression (Onishi et al. 2022), connectivity (Rose and Woolsey 1948; Krettek and Price 1977; Groenewegen 1988; Ray and Price 1992; Vertes 2002; Alcaraz et al. 2016; Pergola et al. 2018; Grodd et al. 2020; Negishi et al. 2024), neurophysiology (Kloet et al. 2020; Lyuboslavsky et al. 2024) and behavior (Vertes et al. 2015; Ouhaz et al. 2018; Wolff and Vann 2018; Kloet et al. 2020; Nelson et al. 2025). Consequently, MD subnuclei have the potential to be differentially affected in and causative of neurological and psychiatric disorders (Popken et al. 2000; Ferguson and Gao 2014; Mitchell et al. 2014; Mitchell 2015; Ouhaz et al. 2018; Consortium et al. 2019). As a major hub for cognitive and social/emotional information, understanding the structural and functional mechanisms specific populations of MD neurons use to filter information will influence how we understand the underlying neurobiology of behavior and design neuromodulation treatments aimed at the prefrontal network (Dembrow and Johnston 2014; Balbinot et al. 2025).

FMRP is encoded by *FMR1*, variants of which can cause fragile X syndrome (FXS), a neurodevelopmental disorder characterized by intellectual disability and autism spectrum disorder. FMRP has cell-type specific effects on the physiology of neurons within and between brain regions (Brager et al. 2012; Routh et al. 2013; Kalmbach et al. 2015). In many *Fmr1*-deficient neurons the physiology of HCN channels is altered. HCN channels are important in regulating cell excitability and rhythmic or bursting activity, and in many cell types are specifically trafficked into dendrites where they modulate synaptic integration. Importantly, the direction of change of HCN channel dysregulation caused by *Fmr1* KO is different across cell types, which results in cell type-specific changes to information processing in KO neurons. In MD, we recently found changes to intrinsic properties and HCN channel activity are subnucleus-specific in MD: MD-L neurons, which express relatively high levels of HCN channel activity have decreased HCN channel activity in the *Fmr1* KO mouse model, while MD-M neurons that have lower baseline HCN channel expression do not show HCN channel changes in *Fmr1* KO mice (Ordemann et al. 2025). FMRP also regulates dendritic arborization and spine maturation in a diverse array of cell types and species (Comery et al. 1997; Lee et al. 2003; Thomas et al. 2008; DeMarco et al. 2022). How loss of FMRP influences dendritic architecture in MD is not understood.

Here, we investigated the dendritic morphology of MD-M and MD-L neurons, specifically those that project to the medial prefrontal cortex (mPFC), in *Fmr1* knockout (KO) mice and their wildtype (WT) littermates (Ordemann et al. 2025). Morphological analyses revealed that MD-M →mPFC and MD-L →mPFC neurons have subtle but consistent differences in the distribution of length and branching in their dendritic arbors. In *Fmr1* KO mice, MD-M →mPFC neurons had an overall decrease in dendritic complexity compared to WT controls. By contrast, no major differences were identified in MD-L →mPFC neurons between groups. Interestingly, these changes in morphology contrast with our physiological findings, which showed that MD-L but not MD-M neurons had decreased HCN channel activity in *Fmr1* KO mice. The uncoupling between *Fmr1*-dependent dendritic morphology and HCN channel function in MD neurons suggests that FMRP regulates structure and function through independent mechanisms in thalamocortical circuits. This circuit-specific pattern of vulnerability has implications for understanding how FXS affects cognitive and social/emotional processing and may inform the development of circuit-targeted therapeutic approaches.

## Methods

### Animals

The animals used in this report are adult mice born in our colony in which *Fmr1* heterozygous (*Fmr1*^-/+^) females (originally a gift of Dr. Kimberly Huber, UT Southwestern to Dan Johnston, UT Austin), congenic to the C57Bl/6J background, were crossed with wildtype C57Bl/6J males from Jackson Labs (stock #000664). A total of 50 mice were used in this study. Our experiments were performed on male mice at 8–12 weeks of age. *Fmr1* KO mice were *Fmr1*^-/y^, WT mice were *Fmr1*^+/y^. Genotyping was performed by Transnetyx. Adult mice were group-housed in open-top cages and fed *ad libitum.* Animals were kept in reverse lighting conditions (8am-8pm dark) and were typically euthanized for experiments in the mornings, around the beginning of the animals’ dark cycle.

### Statistics

We powered the study to detect a difference of 30% in dendritic complexity between wildtype and knockout with a standard deviation of 30% with an alpha of 5% and a beta of 0.08. For a two-tailed t-test, we estimated that we would need at least 16 neurons in each data set. Therefore, we set a sample size goal of at least 20 neurons per data set.

To maximize high quality data collection while keeping animal usage to a minimum, we made electrophysiology recordings from and reconstructed multiple neurons from some mice. Only one neuron per brain slice was quantified. Our data represent *n* = neurons. We reconstructed the dendrites of 50 neurons in WT (MD-M: 26 neurons/26 slices/17 mice, MD-L WT: 24 neurons/24 slices/16 mice) and 53 neurons in KO (MD-M: 26 neurons/26 slices/17 mice; MD-L 27 neurons/27 slices/16 mice).

Principal analyses were performed using GraphPad Prism (version 10.3.1). No cells were excluded from analysis after data analysis began. Data in Table 1 are presented in Figs. 2, 3, 4 as scatter plots with each point representing one neuron. Summary data are medians with 95% confidence intervals. Two-way comparisons were analyzed with Mann–Whitney U test. Data in Table 2 are shown in Figs. 2, 3, 4 as mean ± SEM and were analyzed using 2-way ANOVA with Sidak’s test for multiple comparisons (Table 2). Table 3 data are presented in Fig. 5 data and were analyzed with Spearman correlations. Medians with 95% confidence intervals are also shown.

**Table 1:** Dendrite measurements as median [lower, upper 95% CI] with Mann–Whitney U test

(A) Dendrite measurements as median [95% CI]				
	WT		KO	
	MD-M	MD-L	MD-M	MD-L
Basic dendritic architecture				
Total length (μm)	5677 [5023,6543]	5365 [4308,6504]	4435 [3165,5690]	5250 [4573,6564]
Primary dendrites (#)	5 [4,6]	5 [4,6]	5 [4,5]	5 [5,6]
Nodes (#)	41 [38,52]	44.5 [36,52]	31 [26,41]	45 [40,53]
Terminals (#)	46 [43,55]	49.5 [41,58]	37.5 [31,44]	49 [46,59]
Complexity measures				
Complexity	259,719 [191543,411166]	282,593 [170060,374506]	176,549 [91384,362773]	284,645 [221068,393999]
Spatial extent				
Area (μm^2^)	63726 [54391, 73578]	66006 [56689, 73179]	63863 [52293, 72049]	64682 [54637, 70709]
Volume (μm^3^)	6960240 [5630080, 8780930]	6182040 [4841910, 8930640]	5295295 [4668270, 6806950]	6324680 [5238220, 6957770]

(B) Mann–Whitney U test						
Comparison	WT MD-M vs. MD-L		MD-M: WT vs. KO		MD-L: WT vs. KO	
	*p*	U (95% CI)	*p*	U (95% CI)	*p*	U (95% CI)
Basic dendritic architecture						
Total length (μm)	0.5440	280 (695, 580)	0.0137 *	204 (555, 823)	0.8597	361 (879, 661)
Primary dendrites (#)	0.5700	283.5 (691.5, 583.5)	0.3922	292 (643, 735)	0.2677	267 (759, 567)
Nodes (#)	0.9348	307.5 (658.5, 616.5)	0.0094 **	197.5 (548.5, 829.5)	0.7256	305 (721, 605)
Terminals (#)	0.9501	308.5 (659.5, 615.5)	0.0059 **	189.5 (540.5, 837.5)	0.6906	302.5 (723.5, 602.5)
Complexity measures						
Complexity	0.8249	300 (675, 600)	0.0388 *	225 (576,802)	0.7718	308 (718,608)
Spatial Extent						
Area (μm^2^)	0.8399	301 (652, 623)	0.5309	303 (654, 724)	0.7152	304 (682, 644)
Volume (μm^3^)	0.4016	268 (707, 568)	0.0530	232 (583, 795)	0.8590	314 (692, 634)

**Table 2:** Dendrite Sholl analysis: Two-way ANOVA with Sidak's test for multiple comparisons

Comparison	F value	df	*p* value
Sholl analysis: dendritic length distribution			
WT Comparison (MD-M vs MD-L):			
Subnucleus factor	2.03	1	0.1545
Distance factor	156.7	22	< 0.0001***
Interaction	2.458	22	0.0002**
KO Comparison (MD-M WT vs KO):			
Genotype factor	34.04	1	< 0.0001***
Distance factor	189.5	26	< 0.0001***
Interaction	2.377	26	0.0001***
KO Comparison (MD-L WT vs KO):			
Genotype factor	0.3094	1	0.5782
Distance factor	244	26	< 0.0001***
Interaction	0.07512	26	> 0.9999
Sholl analysis: intersection number			
WT Comparison (MD-M vs MD-L):			
Subnucleus factor	1.411	1	0.2352
Distance factor	156.7	22	< 0.0001***
Interaction	2.679	22	< 0.0001***
KO Comparison (MD-M WT vs KO):			
Genotype factor	29.54	1	< 0.0001***
Distance factor	223.1	26	< 0.0001***
Interaction	1.971	26	0.0025**
KO Comparison (MD-L WT vs KO):			
Genotype factor	0.6543	1	0.4187
Distance factor	232.3	26	< 0.0001***
Interaction	0.07296	26	> 0.9999

Significance levels: *p ≤ 0.05, **p ≤ 0.01, ***p ≤ 0.001

Complete post-hoc comparisons available in supplementary materials

**Table 3:** Voltage sag at -100 mV vs. total dendritic length

	WT		KO	
	MD-L	MD-M	MD-L	MD-M
Spearman r	0.6087	0.1254	0.387	0.1932
95% confidence interval	0.2502 to 0.8204	-0.2951 to 0.5052	-0.03212 to 0.6903	-0.2214 to 0.5486
*P* (two-tailed)	0.0021	0.5503	0.0618	0.3444
*P* value summary	**	ns	ns	ns
Exact or approximate *P* value?	Approximate	Approximate	Approximate	Approximate
Significant? (alpha = 0.05)	Yes	No	No	No
Number of XY Pairs	23	25	24	26

We also ran our statistics using a linear mixed model (R version 4.4.2) to account for mouse and slice as factors and found they did not contribute to pseudoreplication (Hurlbert 1984) (see Supplemental Materials).

### Masking of group identities

The person (P.L.) performing the electrophysiological recordings by necessity knew the location of each cell at the time of recording. The scientist (A.K.) who prepared the brain slices for histology did not know the identify (MD-M vs. MD-L or *Fmr1* wildtype vs. knockout) of the cells during the preparation of the tissue or during tracing of the dendrites.

### Fluorescent labeling of MD →mPFC neuron populations

Mice were anesthetized with 2% isoflurane and mounted in a stereotaxic frame. Craniotomies were made according to stereotaxic coordinates relative to Bregma. To label mediodorsal thalamus (MD) neurons that project to ipsilateral prelimbic and infralimbic cortices (ipsilateral mPFC), we injected fluorescently-labeled cholera toxin subunit B (CTB, 500 µg/100 µL, Molecular Probes, Thermo Fisher Scientific, Waltham, MA, USA) into ipsilateral mPFC (Nanofil Syringe and Pump UMP3, World Precision Instruments). Coordinates for injection into ipsilateral mPFC were (in mm relative to Bregma): –1.7 anterior–posterior (AP), + 0.3 mediolateral (ML) and –2.75 dorsoventral (DV). After needle insertion, we waited 5 min before starting the injection of 450 nL at 100 nL/minute into mPFC. We waited 5 min after the end of the injection before slowly withdrawing the syringe needle. We waited 3–6 days following retrograde tracer injections before performing experiments. At the time of the experiments, we visually verified that retrograde tracer injections were targeted appropriately, and that tracer was not present in nearby structures. All cells reported here are retrogradely-labeled MD-L →mPFC or MD-M →mPFC neurons; “MD-M” and “MD-L” are used in the manuscript for brevity.

### Slice preparation

Except as noted, all reagents for patch clamp electrophysiology solutions were purchased from Sigma Aldrich. We used ice-cold cutting solution containing (in mM): 205 sucrose, 25 NaHCO_3_, 2.5 KCl, 1.25 NaH_2_PO_4_, 7 MgCl_2_, 7 dextrose, 3 Na pyruvate, 1.3 sodium ascorbate, and 0.5 CaCl_2_ bubbled with 95% O_2_/5% CO_2_. Mice 8 to 12 weeks old were deeply anesthetized with intraperitoneal injection of ketamine/xylazine (90/10 mg/kg; Acor/Dechra), transcardially perfused with 20 mL of ice-cold cutting solution, and then decapitated. We prepared 300 µm coronal slices (Leica VT1200). Slices were incubated in holding solution containing (in mM): 125 NaCl, 25 NaHCO_3_, 2.5 KCl, 1.25 NaH_2_PO_4_, 25 dextrose, 2 CaCl_2_, 2 MgCl_2_, 1.3 sodium ascorbate, and 3 Na pyruvate at 37 ± 1°C for 30 min. Slices were then kept for at least 30 min at room temperature before being used for recordings. We typically obtained 3 MD thalamic slices per brain.

### Intracellular recordings

Artificial cerebrospinal fluid (ACSF) contained (in mM): 125 NaCl, 25 NaHCO_3_, 12.5 dextrose, 2.5 KCl, 1.25 NaH_2_PO_4_ 2 CaCl_2_ and 1 MgCl_2_. Slices were continuously perfused with ACSF in an immersion chamber (Warner Instruments) with temperature maintained at 32.5 ± 1°C (Warner Instruments TC-324C). We did not add synaptic blockers to the ACSF unless otherwise specified.

Somatic whole-cell patch recordings were obtained from visually-identified retrogradely-labeled neurons in the medial (MD-M) or lateral (MD-L) subnuclei, using DODT (Zen 2.5 blue addition, Zen pro) contrast microscopy and epifluorescence on an upright microscope (Zeiss Examiner D1). Patch electrodes (tip resistance = 3–6 MΩ) were filled with the following (in mM): 118 K-gluconate, 10 KCl, 10 HEPES, 4 MgATP, 1 EGTA, 0.3 Na3GTP and 0.3% biocytin (pH adjusted to 7.2 with KOH; 282 mOsM).

### Fixed tissue processing

After recording, slices were stored overnight at 4°C in 4% PFA (Electron Microscopy Science) and moved to phosphate-buffered saline (PBS) the following morning. Within 60 days, we stained cells with DAB to reveal biotin filling. First, we washed slices in 0.1M PBS for 30 min (6 × 5 min) with the consecutive washing in 1% Triton (Fisher Chemical) and 0.5% H_2_O_2_ (Fisher Chemical) solutions in PBS for 30 min each at room temperature (RT). After washing in 0.1M PBS again for 30 min, we incubated slices in Reagent A + Reagent B + 0.5% TritonX + 0.1% BSA (Sigma-Aldrich) in PBS for 2 days at 4^o^C (ABC reagent, VectaStain Elite ABC-HRP kit, Vector laboratories). After 2-day incubation, we washed slices in PBS for an hour (6 × 10 min). Then we incubated slices with 3,3′-diaminobenzidine (DAB substrate kit, Vector laboratories) solution for one hour at RT. After that, we added 3% H_2_O_2_ to the slices (5 drops every 20 min) until they reached the desirable brown color. Then we washed slices in PBS again for 30 min and dehydrated them in ascending concentrations of glycerol (Fisher Chemical) from 20 to 100% for 10 min per concentration. We mounted slices on slides using 100% glycerol as the mounting medium. 

### Quantification of dendritic arbors

We reconstructed cells using the Neurolucida System (MBF Bioscience Inc. Williston, VT) on Leitz Diaplan microscope and MicroFire Optronics camera using a 100× objective. We placed the zero point in the center of the soma (at the widest part of the soma) and traced dendrites using a fine focusing wheel. We considered branches as cut when they visibly went out of the slice. We considered cells usable for analysis if they had fully-visualized dendritic arbors with well-defined terminals and with most branches being present. For the cells with 1 or 2 cut dendrites (close to the cell body), we present the analysis with those dendrites excluded. We partially reconstructed axons from a subset of neurons in each group (shown in red).

### Histology

To create an atlas of fixed brain slice images onto which we could map all recorded cells, we injected an 11-week-old C57Bl/6 wildtype mouse with CTB into the prelimbic and infralimbic cortices as above. The animal was deeply anesthetized with ketamine/xylazine and transcardially perfused with paraformaldehyde (PFA (Sigma-Aldrich)) 4% in phosphate-buffered saline (1× PBS). We prepared 50 µm thick coronal slices with DAPI-containing mounting medium (VectaShield HardSet with DAPI, Vector laboratories). We imaged the slices at 5× using Zeiss Axio Imager 2 and stitched images together using AxioVision software.

### Sholl analysis

We used Neurolucida Explorer (MBF Bioscience Inc. Williston, VT) to analyze the cell's morphological parameters. We measured dendritic length (summed length of all branches of the neuron (µm)), total number of primary dendrites, branching points (nodes) per cell and total number of last order branches (terminals) per cell. We used convex hull analysis to measure area (µm^2^) and volume (µm^3^). These parameters correspond to the area and volume of a convex polygon that connects tips of distal processes. For Sholl analysis (Sholl 1953), we placed concentric circles in 15 µm radius increments around the cell centered at the cell body. We counted the number of points where cell processes intersect with Sholl circles. Within each circle, we also measured dendritic length.

We calculated cellular complexity using the following formula:

$$ Complexity = \left( {sum\;of\;ter\min al\;orders + \# \;of\;ter\min als} \right) \cdot \frac{total\;dendritic\;length}{{\# \;of\;primary\;dendrities}} $$

For each terminal in a cell, the *terminal order* is the total number of sister branches encountered a path is traced from that terminal to the cell body. The *sum of terminal orders* is the sum of all *terminal order*s for each cell.

## Results

**Fig. 1:**
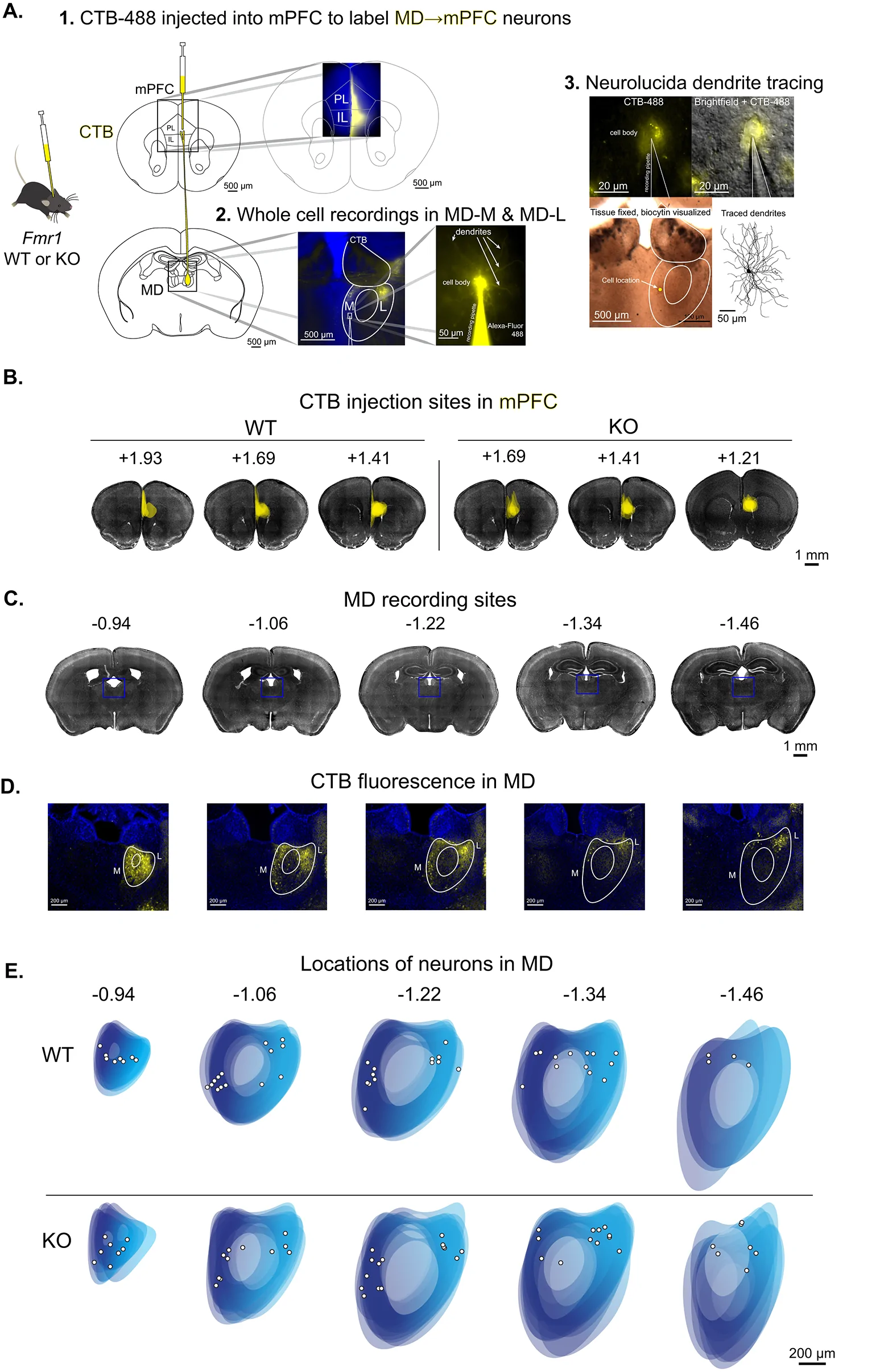
Retrograde labeling and identification of MD →mPFC projection neurons. **A** Experimental workflow. **1.** Fluorescently-labeled cholera toxin subunit B (CTB-488) was injected into the medial prefrontal cortex (mPFC) of wildtype (WT) or *Fmr1* knockout (KO) mice to retrogradely label mediodorsal thalamus (MD) neurons projecting to mPFC. **2.** After 3–6 days, acute brain slices were prepared and whole-cell recordings were obtained from retrogradely-labeled MD-M and MD-L neurons. Representative images show CTB fluorescence in MD (left), with medial (M) and lateral (L) subnuclei outlined, and cell body with biocytin filling during recording (right). **3.** Following recordings, tissue was fixed and processed for dendrite tracing and quantification. Top panels show a representative neuron labeled with CTB-488. In the bottom left panel, the yellow dot shows the location of the recorded neuron in fixed tissue. The bottom right panel shows its traced dendrites. **B** CTB injection sites in mPFC for WT (left) and KO (right) mice. Coronal sections shown at indicated distances (mm) anterior to Bregma with distributions of CTB injections overlayed in yellow. **C.** Recording sites within MD thalamus. Coronal sections shown at indicated distances (mm) posterior to Bregma. Scale bar: 1 mm. **D** CTB fluorescence in MD showing retrogradely-labeled neurons from boxed regions of (**C**). Representative images from five anterior-posterior levels showing CTB-positive neurons (yellow) and DAPI nuclear staining (blue). Labeled cells were mostly found in the medial (MD-M) and lateral (MD-L) subnuclei. The central subnucleus, MD-C, was largely unlabeled by CTB injected into mPFC. Scale bars: 200 μm. **E** Three-dimensional reconstructions of MD thalamus showing spatial distribution of all recorded and reconstructed neurons. Each white dot represents one neuron. Reconstructions shown for WT (top row) and KO (bottom row) mice across five anterior-posterior positions (indicated in mm posterior to Bregma). The medial-to-lateral gradient of the thalamus is represented by the blue color gradient, with darker blue indicating medial regions (MD-M) and lighter blue indicating lateral regions (MD-L). This color scheme is used throughout the paper to denote MD-M →mPFC and MD-L →mPFC neurons. Scale bar: 200 μm

Dendrites are the primary neuronal structures that process incoming synaptic information. The structure of the dendritic arbor influences the integration and interpretation of synaptic activity. Thus, the dendritic architecture of MD neurons influences the gating of information destined for the medial prefrontal cortex (mPFC) and other brain regions. To isolate MD neurons that project to mPFC, we injected the fluorescently-labeled retrograde tracer CTB into unilateral prelimbic and infralimbic cortex of adult mice (Fig. 1). After 3–6 days, we prepared acute *ex vivo* coronal brain slices. We obtained whole-cell physiology recordings of labeled MD neurons ipsilateral to the injection site and included biocytin in the intracellular solution (Ordemann et al. 2025). After recordings, we fixed the brain slices and processed them to visualize biocytin. We then traced the dendritic arbors and quantified dendritic morphology as illustrated in Fig. S3. See Tables 1 and 2 for full statistics.

**Baseline differences between MD-M and MD-L neurons in wildtype mice.** While MD-M and MD-L both project to mPFC, accumulating evidence suggests they have distinct roles in behavior. To understand if MD-M and MD-L neurons also have differences in subcellular structure, we first characterized their baseline morphological and functional properties in wildtype (WT) mice.

**Fig. 2:**
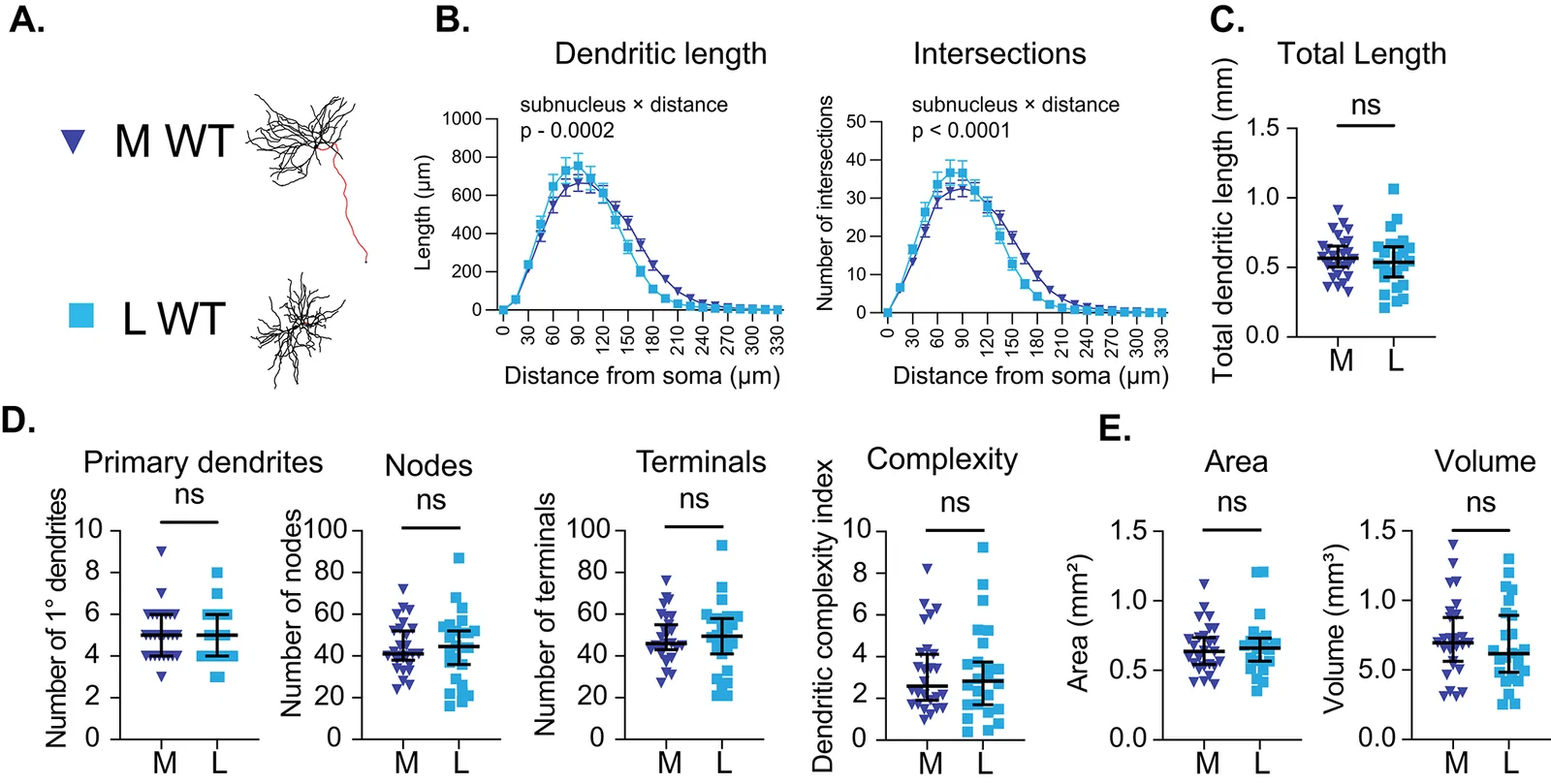
MD-M neurons show more distal dendritic branching than MD-L neurons. **A** Representative neurons from MD-M and MD-L of wildtype mice. Dendrites are black. Axons are red. Cut dendrites denoted by filled circles. **B** Sholl plots of dendritic length and number of intersections show a distal shift in MD-M neurons (Length: subnucleus *p* = 0.1545, interaction of distance × subnucleus *p* = 0.0002; Intersections: subnucleus *p* = 0.2352, interaction of distance × subnucleus p < 0.0001). **C.** MD-M and MD-L neurons had similar total dendritic length (*p* = 0.5440). **D.** MD-M and MD-L neurons did not differ in number of primary dendrites (*p* = 0.5700), dendritic nodes (*p* = 0.9348), or terminals (*p* = 0.9501). MD-M and MD-L neurons had similar complexity (*p* = 0.8249). **E** MD-M and MD-L neurons had similar field area (*p* = 0.8399) and volume (*p* = 0.4016). Sholl plots show mean ± standard error of the mean. Scatter plots show all cells as individual data points and median ± 95% Cl. Significance is indicated as not significant (ns; *p* > 0.05), or significant at *p* < 0.05 (*). For **B:** Sidak's test. For **C**, **D**, **E** two-tailed Mann–Whitney

*Morphological differences.* Sholl analysis quantified by two-way ANOVA, corrected for multiple comparisons, revealed significant differences in the arborization of dendrites between MD-M and MD-L neurons. The distribution of dendritic length (2-way ANOVA interaction of subnucleus × distance from soma, p = 0.0002) and number of intersections (i.e., number of dendritic branches; 2-way ANOVA interaction of subnucleus × distance from soma, p < 0.0001) are shifted distally in MD-M neurons compared with MD-L (Fig. 2B**, **Table 2). Despite this difference in distribution, the total dendritic length was unchanged between the two subnuclei (Fig. 2C, p = 0.5440). There were no differences in the number of primary dendrites (*p* = 0.5700), nodes (*p* = 0.9348), or terminals (*p* = 0.9501). There was no difference between MD-M and MD-L in overall dendritic complexity (p = 0.8249; Fig. 2D), a quantification of “branchiness” and is a function of the total dendritic length, number of primary dendrites, nodes, and terminals (**Methods**). Neither area (*p* = 0.8399) nor the volume (*p* = 0.4016) was significantly different between groups (Fig. 2E). These data suggest that while the dendritic arbors of WT MD-M and MD-L neurons take up similar space, with similar amounts of dendritic surface, there is a subtle but significant difference in how the branching dendrites are distributed within the arbor.

*Functional differences.* In addition to these morphological differences, we have previously demonstrated that MD-M and MD-L neurons differ in their intrinsic electrophysiological properties (Lyuboslavsky et al. 2024; Ordemann et al. 2025). Specifically, MD-L neurons exhibit significantly higher HCN channel activity than MD-M neurons. This difference in HCN channel function has important implications for information processing: MD-M neurons, with relatively low HCN channel activity, have higher membrane resistance and longer time constants, allowing them to integrate synaptic inputs more readily over time and function as sensitive detectors of weak or asynchronous inputs. By contrast, MD-L neurons, with higher HCN channel activity, have reduced input resistance and shorter membrane time constants, making them function as coincidence detectors that require either larger synaptic inputs or well-synchronized smaller inputs to reach action potential threshold. Thus, the morphological difference in distal dendritic branching in MD-M may complement their functional properties to enhance sensitivity to distal synaptic and neuromodulatory inputs, while the more proximal branching pattern in MD-L aligns with their role in specific, synchronized information processing.

**Loss of *Fmr1* produces circuit-specific structural changes.** Having established baseline differences between MD-M and MD-L in WT mice, we next asked whether loss of *Fmr1* affects dendritic morphology in these two subnuclei. Since *Fmr1* loss leads to differential changes in dendritic and spine morphology across different neuron types, we hypothesized that the effects on MD subnuclei might also be circuit-specific. We measured and compared dendritic arborization in wildtype and *Fmr1* knockout (KO) littermates in both MD-M and MD-L neurons.

**Fig. 3:**
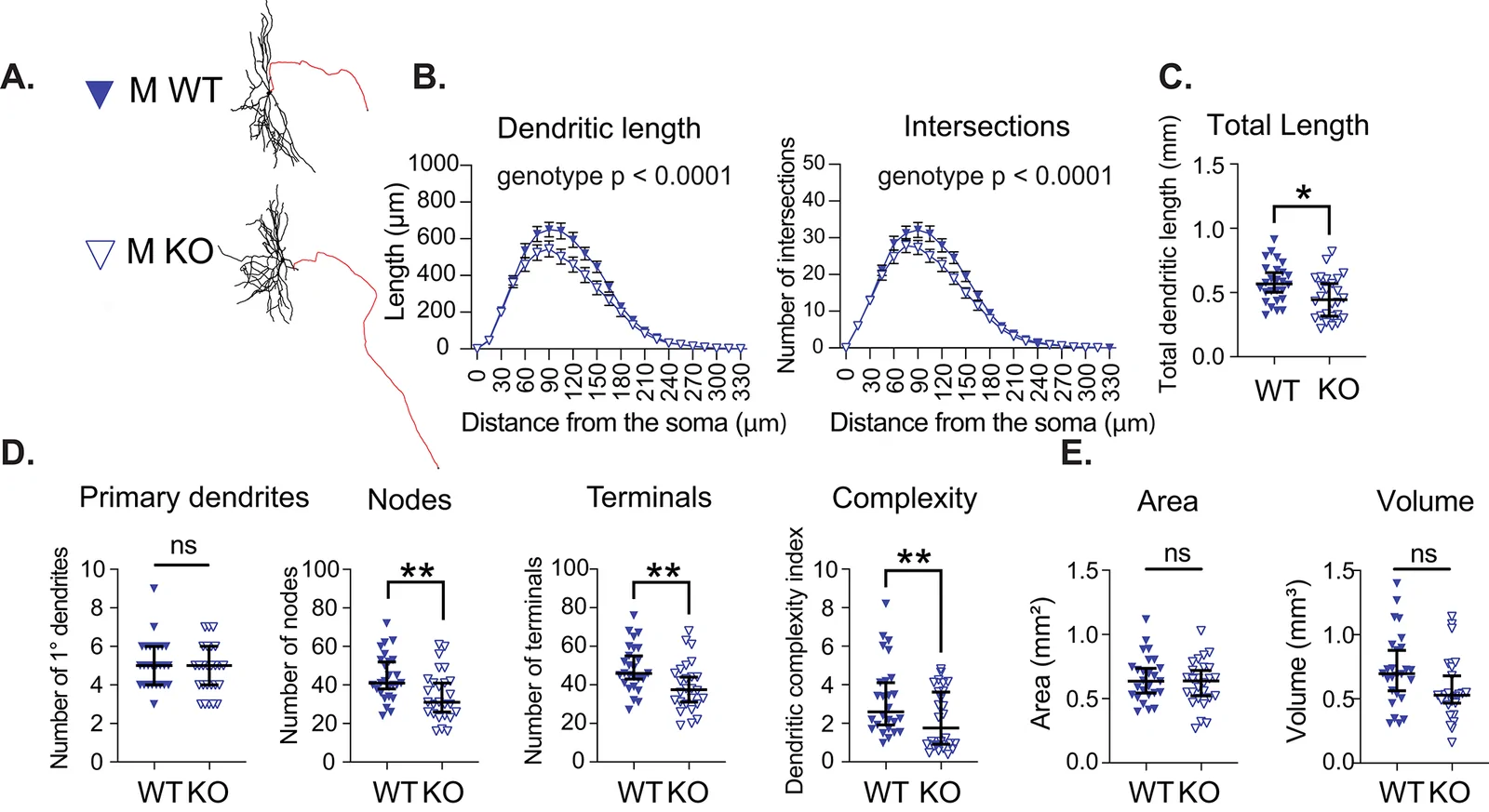
Loss of *Fmr1* decreases dendritic complexity in MD-M neurons. **A** Representative neurons from MD-M neurons in Fmr1 wildtype and knockout mice. Dendrites are black. Axons are red. Cut dendrites denoted by filled circles. **B** Sholl plots of dendritic length and number of intersections decreased in *Fmr1* KO animals (Length: genotype *p* < 0.0001, interaction distance × subnucleus *p* = 0.0001; Intersections: genotype p < 0.0001, interaction distance × subnucleus *p* < 0.0025). **C.** *Fmr1* KO neurons had decreased total dendritic length (*p* = 0.0137). **D** *Fmr1* KO neurons did not differ in number of primary dendrites (*p* = 0.3922) but had decreased numbers of dendritic nodes (*p* = 0.0094) and terminals (*p* = 0.0059). *Fmr1* KO neurons had decreased complexity (*p* = 0.0388). **E** Fmr1 KO neurons had similar field area (*p* = 0.5309) and volume (*p* = 0.0530). Sholl plots show mean ± standard error of the mean. Scatter plots show all cells as individual data points and median ± 95% Cl. Significance is indicated as not significant (ns; *p* > 0.05), or significant at *p* < 0.05 (*), *p* < 0.01 (**). For **B** Sidak's test. For **C**, **D**, **E** two-tailed Mann–Whitney

*Loss of Fmr1 decreases dendritic complexity in MD-M.* Sholl analysis revealed decreased dendritic length (2-way ANOVA genotype factor, *p* < 0.0001) and number of intersections (2-way ANOVA genotype factor, p < 0.0001) in MD-M neurons from *Fmr1* KO mice compared to WT littermates (Fig. 3B**, **Table 2). Total dendritic length was decreased (Fig. 3C; Mann–Whitney, *p* = 0.0137) in *Fmr1* KO mice. There were no differences in the number of primary dendrites (p = 0.3922), but the number of nodes (Mann–Whitney, p = 0.0094) and terminals (Mann–Whitney, *p* = 0.0059) were decreased (Fig. 3D). Overall, dendrites in the *Fmr1* KO mice were less complex (Mann–Whitney, *p* = 0.0388) compared to wildtype littermates. This decreased dendritic complexity was not accompanied by changes in area (Mann–Whitney, *p* = 0.5309) or volume (Mann–Whitney, *p* = 0.0530) taken up by the dendritic arbors (Fig. 3E). Together, these suggest that while *Fmr1* KO MD-M neurons have similar numbers of primary dendrites and extend over similar areas, their arbors are less “branchy” and have less dendritic surface area upon which to form synapses.

**Fig. 4:**
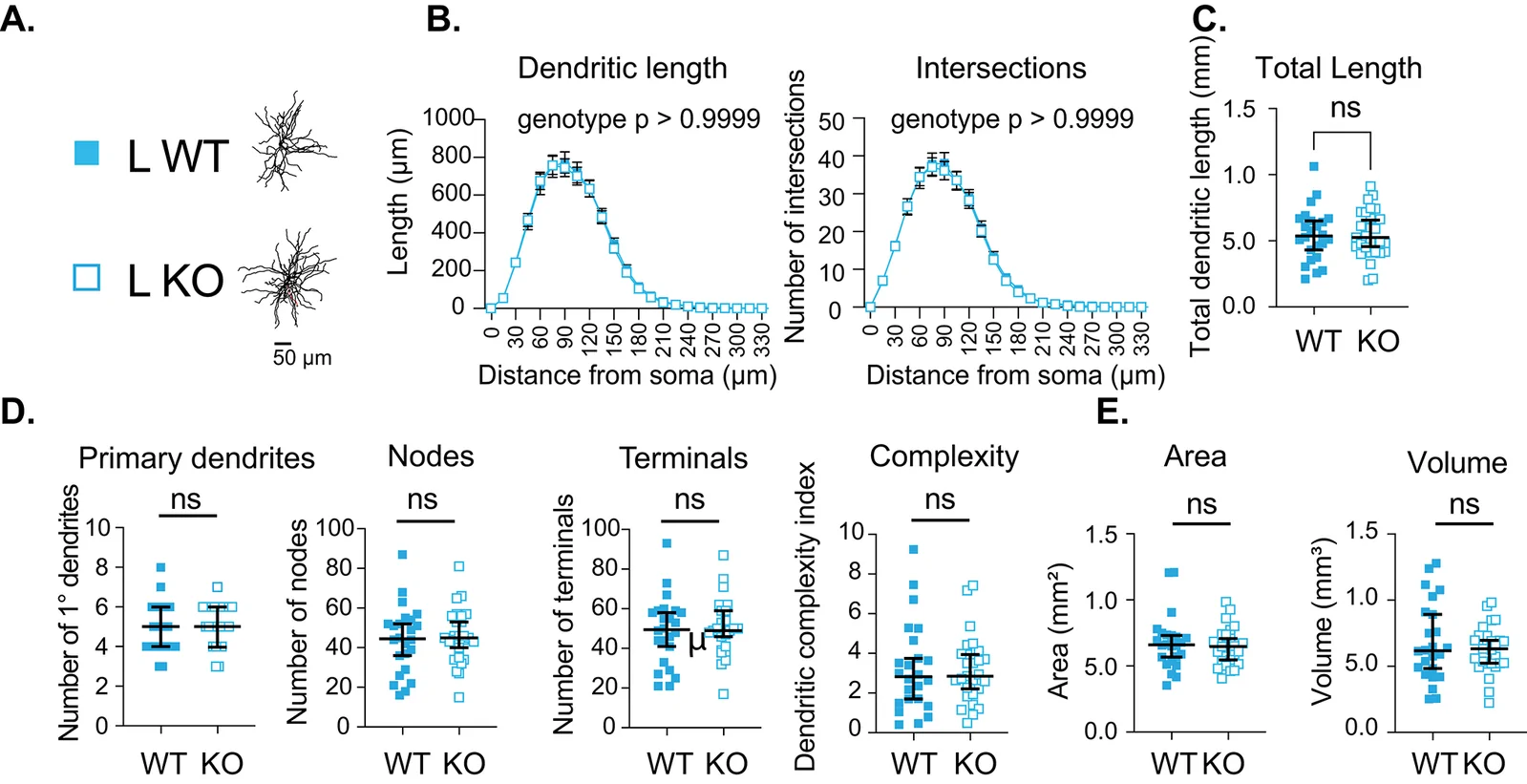
Loss of *Fmr1* does not affect dendritic complexity in MD-L neurons.** A** Representative neurons from MD-L neurons in *Fmr1* wildtype and knockout mice. **B** Sholl plots of dendritic length and number of intersections are not different in *Fmr1* KO animals (Length: subnucleus *p* > 0.9999, interaction of distance x subnucleus *p* = 0.5782; Intersections: subnucleus *p* = 0.4187, interaction of distance x subnucleus *p* > 0.9999). **C** *Fmr1* KO neurons had normal total dendritic length (*p* = 0.8597) **D.** *Fmr1* KO neurons did not differ in number of primary dendrites (*p* = 0.2677), dendritic nodes (*p* = 0.7256), or terminals (*p* = 0.6906). *Fmr1* KO neurons had normal dendritic complexity (*p* = 0.7718). **E** *Fmr1* KO neurons had similar field area (*p* = 0.7152) and volume (*p* = 0.8590). Sholl plots show mean ± standard error of the mean. Scatter plots show all cells as individual data points and median ± 95% Cl. Significance is indicated as not significant (ns; *p* > 0.05). For **B** Sidak's test. For **C D**, **E** two-tailed Mann–Whitney

*Loss of Fmr1 does not affect dendritic complexity in MD-L.* While MD-M dendritic morphology was influenced by loss of *Fmr1*, our analyses of MD-L neurons revealed that their dendrites were unchanged. Sholl analysis revealed similar total dendritic length (2-way ANOVA, genotype factor, *p* = 0.5782) and number of intersections (genotype factor, *p* = 0.4187) in MD-L neurons in *Fmr1* KO mice compared with WT littermates (Fig. 4B**, **Table 2). There was no difference in total length (Mann–Whitney, *p* = 0.8597; Fig. 4C). There were no differences in the number of primary dendrites (Mann–Whitney, *p* = 0.2677), nodes (Mann–Whitney, *p* = 0.7256), or terminals (Mann–Whitney, *p* = 0.6906), and no change in dendritic complexity (Mann–Whitney, *p* = 0.7717; Fig. 4D). Neither the area (Mann–Whitney, p = 0.7152) nor the volume (Mann–Whitney, p = 0.8590) was significantly different between groups (Fig. 4E). These data show a selective vulnerability of MD-M neuron structure in *Fmr1* KO model mice that is not observed in MD-L neurons.

**Dissociation between morphological and functional changes in *Fmr1* KO mice.** Our morphological data reveal that MD-M neurons exhibit structural changes in *Fmr1* KO mice, while MD-L neurons do not. Intriguingly, our previous physiological studies show the opposite pattern: MD-L neurons, but not MD-M neurons, exhibit altered HCN channel activity in *Fmr1* KO mice (Ordemann et al. 2025). This suggests a striking dissociation between structural and functional phenotypes.

To further explore the relationship between dendritic structure and HCN channel function, we examined correlations between voltage sag (a physiological measurement of HCN channel function) and total dendritic length for each neuron. In pyramidal neurons, HCN channels have a gradient of increasing expression toward the distal end of the apical dendrite (Lörincz et al. 2002). For thalamic neurons, which have multiple primary dendrites, it is unknown how HCN channels are distributed.

**Fig. 5:**
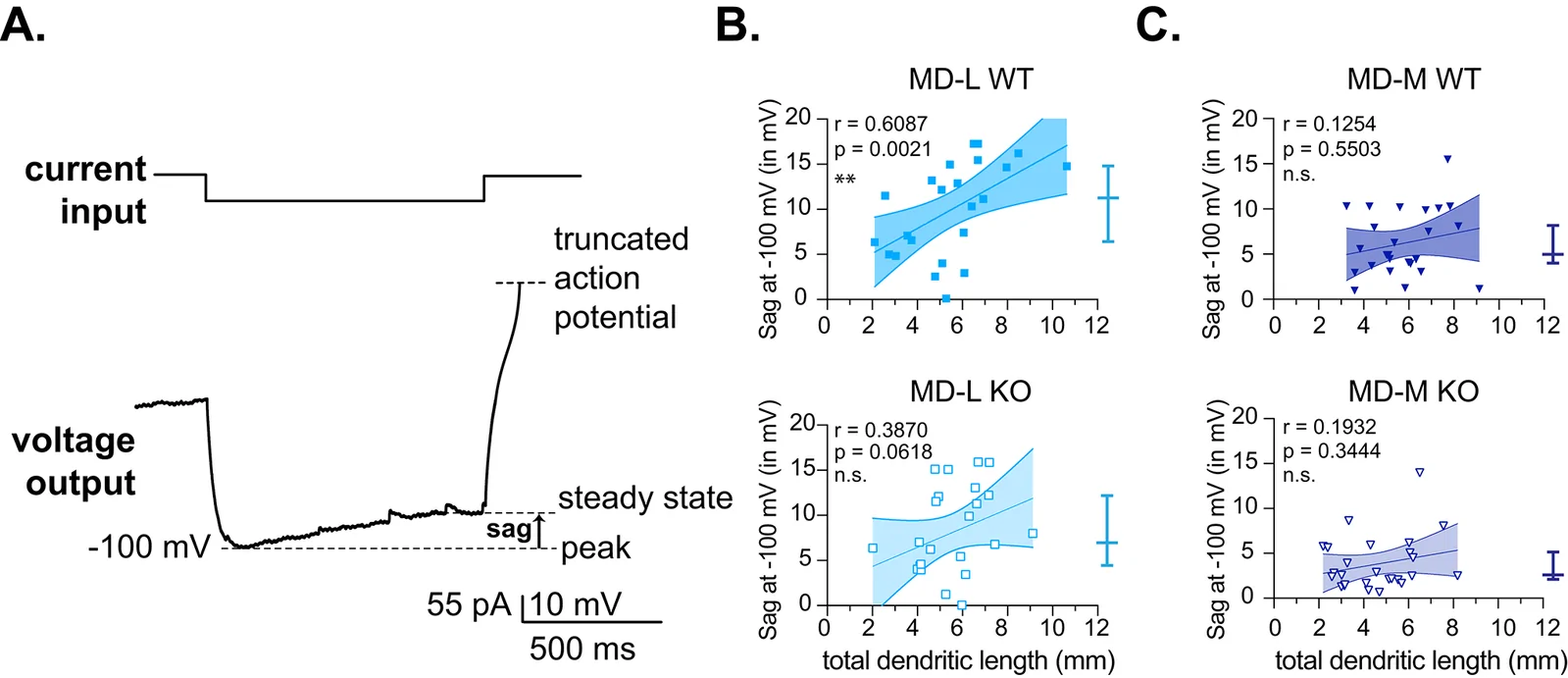
Dissociation between HCN channel activity and dendritic morphology in MD subnuclei. **A** Schematic illustrating voltage sag measurement protocol. Membrane potential was stepped to -100 mV with hyperpolarizing current injection (top trace). Voltage sag reflects HCN channel activity and is quantified as the difference between the peak hyperpolarization and the steady-state voltage (bottom trace). Scale bars: 55 pA, 10 mV, 500 ms. **B** Correlation between voltage sag and total dendritic length in MD-L neurons from wildtype (WT, top) and *Fmr1* knockout (KO, bottom) mice. Each point represents an individual neuron (WT: *n* = 24; KO: *n* = 27). Solid lines indicate linear regression fit with 95% confidence intervals (shaded regions). Insets show median ± 95% confidence intervals for voltage sag. In WT mice, MD-L neurons exhibit a significant positive correlation between sag and dendritic length (*r* = 0.6087, *p* = 0.0021, **p < 0.01). In *Fmr1* KO mice, this correlation is weakened and no longer significant (*r* = 0.387, *p* = 0.0618, n.s.). **C.** Correlation between voltage sag and total dendritic length in MD-M neurons from WT (top) and KO (bottom) mice. Each point represents an individual neuron (WT: *n* = 26; KO: *n* = 26). Solid lines indicate linear regression fit with 95% confidence intervals (shaded regions). Insets show median ± 95% confidence intervals for voltage sag. MD-M neurons show no significant correlation between sag and dendritic length in either WT (*r* = 0.1254, *p* = 0.5503, n.s.) or KO (*r* = 0.1932, *p* = 0.3444, n.s.) mice. Full statistical analysis is provided in Table 3

In wildtype mice, MD-L neurons displayed a strong positive correlation between voltage sag and dendritic length (Spearman *r* = 0.6087, *p* = 0.0021; Fig. 5), consistent with increased HCN expression in longer dendritic arbors. This correlation was weakened in *Fmr1* KO animals (Spearman *r* = 0.387, *p* = 0.0618), potentially reflecting the disruption of HCN channel regulation. By contrast, MD-M neurons showed no relationship between voltage sag and total dendritic length in either WT (Spearman *r* = 0.1254, *p* = 0.5503) or *Fmr1* KO animals (Spearman *r* = 0.1932, *p* = 0.3444).

These data reveal a striking dissociation between structural and functional phenotypes in *Fmr1* KO mice: MD-M neurons exhibit altered dendritic morphology without changes in HCN channel physiology, whereas MD-L neurons show dysregulated HCN channel activity without morphological changes. This double dissociation suggests that FMRP regulates structure and function through independent mechanisms in these two thalamocortical circuits. The independence of these regulatory pathways may have important implications for understanding the diverse cellular consequences of FMRP loss and for developing circuit-specific therapeutic strategies in fragile X syndrome.

## Discussion

In this study, we characterized the dendritic morphology of mediodorsal thalamus (MD) neurons projecting to the medial prefrontal cortex (mPFC) in wildtype and *Fmr1* knockout mice. We report three main findings. First, in wildtype mice, MD-M and MD-L neurons exhibit subtle but consistent differences in dendritic arborization, with MD-M neurons showing a more distal distribution of dendritic branching compared to MD-L neurons. Second, loss of *Fmr1* produces circuit-specific structural changes: MD-M neurons show decreased dendritic complexity in *Fmr1* KO mice, while MD-L neurons exhibit no morphological changes. Third, we find a dissociation between structural and functional phenotypes across subnuclei: MD-M neurons were structurally vulnerable to *Fmr1* loss, whereas MD-L neurons were functionally vulnerable. This dissociation suggests that FMRP regulates dendritic morphology and ionic conductances through independent mechanisms in different thalamocortical circuits.

**Structural differences complement functional specialization in MD subnuclei.** The baseline morphological difference we observed between MD-M and MD-L neurons—a more distal distribution of dendritic branching in MD-M—may contribute to the functional specialization of these subnuclei. MD thalamus projects to mPFC and other brain regions involved in cognitive and social/emotional behaviors related to autism and intellectual disability (Vertes et al. 2015; Parnaudeau et al. 2017). The medial and lateral subregions of MD differ in their connectivity, activation patterns, and electrophysiological properties (Frith and Frith 2006; Mátyás et al. 2014; Hunnicutt et al. 2014; Vertes et al. 2015; Alcaraz et al. 2016; Bolkan et al. 2017; Ährlund-Richter et al. 2019; Lyuboslavsky et al. 2024), consistent with distinct roles: MD-M has been hypothesized as the social/emotional or limbic subdivision of MD thalamus, whereas MD-L may play a relatively stronger role in cognitive processes (Vertes et al. 2015; Wolff and Vann 2018; Grodd et al. 2020).

Our previous work demonstrated that differences in intrinsic physiological properties optimize these subnuclei for different information processing modes: MD-M neurons are relatively 'sensitive', whereas MD-L neurons are more 'specific' (Lyuboslavsky et al. 2024). With relatively low baseline HCN channel function, MD-M neurons have higher membrane resistance and thus longer time constants, allowing them to integrate synaptic inputs more readily over time. With a long integration window, small and asynchronous synaptic inputs can trigger action potentials in MD-M neurons, making them sensitive to inputs. By contrast, MD-L neurons, with relatively increased HCN channel activity that reduces input resistance and shortens membrane time constants, filter out extraneous information by requiring either larger synaptic inputs or well-synchronized smaller inputs to reach action potential threshold, effectively functioning as coincidence detectors.

The morphological differences we report here may enhance these functional specializations. MD-M neurons exhibited a modest shift towards more distal branching compared to MD-L neurons. This distal branching could increase the relative contribution of distal synaptic and neuromodulatory inputs to somatic signaling (Makarov et al. 2023). Combined with relatively long integration time windows, this morphological specialization may enhance the responsivity of MD-M neurons to weak distal dendritic inputs, thus making them more sensitive, while the proximal-shifted branching of MD-L would make them more specific.

**Circuit-specific structural vulnerability to *Fmr1* loss.** People with fragile X syndrome (FXS) exhibit changes in both social/emotional and cognitive processing, and dysfunction in the *Fmr1* gene is linked to both physiological and morphological changes in neurons (Hagerman et al. 2017; Mila et al. 2018). Our recent work demonstrated that loss of *Fmr1* in MD-L neurons, but not MD-M neurons, decreased HCN activity and delayed spike timing (Ordemann et al. 2025). Here we show the opposite pattern for dendritic morphology: loss of *Fmr1* selectively decreased the dendritic complexity of MD-M but not MD-L neurons. This double dissociation—morphological changes in MD-M without functional changes, functional changes in MD-L without morphological changes—is particularly striking because both phenotypes are caused by loss of the same gene in closely related thalamocortical circuits.

How *Fmr1* influences dendritic morphology is variable, depending on the species, cell type, and developmental stage studied. Some reports demonstrate that loss of *Fmr1* decreases the complexity of dendrites in FXS patient-derived neurons (Guo et al. 2023), mouse dentate granule neurons (Shen et al. 2019; Yau et al. 2019), zebrafish tectum pyramidal neurons (DeMarco et al. 2022), chicken nucleus magnocellularis neurons (Wang et al. 2018), and *Drosophila* larvae “dendritic arborization neurons” (Lee et al. 2003). Other studies have shown that the absence of *Fmr1* *increased* dendritic growth and complexity in mouse hippocampal dentate granule neurons (Sathyanarayana et al. 2022), CA3 pyramidal neurons (Scharkowski et al. 2018), and retinal ganglion cells (Vlasits et al. 2024). It has also widely been reported that loss of *FMR1* function influences dendritic spine morphology in humans (Rudelli et al. 1985; Hinton et al. 1991; Irwin et al. 2001) and in animal models of FXS (Galvez et al. 2003; Grossman et al. 2006).

Our findings of decreased dendritic complexity in MD-M without changes in MD-L add to this literature by demonstrating circuit-specific vulnerability within a single brain region. This circuit-level specificity in the function of *Fmr1* may reflect differences in the local molecular environment, developmental timing, or the repertoire of FMRP target mRNAs expressed in these two populations.

*Fmr1*-associated decreases in dendritic length in MD-M could have complex impacts on information processing. Lower dendritic surface area would decrease the space for, and thus potentially the number of, synapses onto MD-M neurons. Less dendritic surface area would also decrease dendritic membrane capacitance, shortening the temporal window during which synaptic inputs onto MD-M dendrites can summate. Fewer synaptic inputs and a shorter dendritic membrane time constant could decrease the sensitivity of MD-M cells. In addition to decreased total length, MD-M neurons in *Fmr1* KO mice had decreased branching. Decreased branching could further decrease synaptic integration by decreasing the dynamic range of input–output functions (Ferrante et al. 2013; Lin et al. 2023). MD-M receives inputs from brain regions important for social/emotional functioning such as the amygdala and insula (Georgescu et al. 2020; Timbie et al. 2020). Decreased complexity of MD-M dendrites alone could therefore lead to overall decreased transmission of affective signals to downstream targets like the prefrontal cortex. It is also possible that decreased complexity in MD-M neurons could *increase* the relative amplitude and reliability of distal synaptic events affecting somatic membrane potential (Poirazi and Papoutsi 2020; Sinha and Narayanan 2022). Therefore, in the absence of *Fmr1*, MD-M neurons may become even more sensitive to dendritic synaptic inputs such that the prefrontal cortex receives a higher level of background activity compared to wildtype. This could worsen the signal-to-noise ratio for social/emotional signaling and result in higher baseline anxiety, as is seen in people with FXS.

**Limitations and considerations.** Our study has several important limitations that should be considered when interpreting these findings. First, all neurons reported here were reconstructed from 300 μm thick brain slices, and only neurons near the surface (suitable for whole-cell physiology) were included. Thus, our measurements do not represent the complete three-dimensional dendritic arbor present in the intact brain, and some distal dendrites may have been truncated during slicing. However, this limitation applies equally to all groups, and the systematic differences we observe between genotypes are unlikely to be artifacts of the slicing procedure. Second, our approach did not provide sufficient optical resolution to analyze dendritic spine morphology, which is known to be affected by *Fmr1* loss in many brain regions. Future studies using higher resolution microscopy or alternative labeling approaches will be needed to assess spine changes in MD. Third, we recorded only MD neurons that project to mPFC; whether MD neurons projecting to other brain regions have similar morphological profiles remains to be determined. Fourth, consistent with the overwhelming male bias in FXS diagnosis, we analyzed only male mice. While females with full *FMR1* mutations can develop FXS (Bartholomay et al. 2024), future studies should include female subjects to determine if sexual dimorphism affects these phenotypes. Finally, it remains unclear whether the dendritic changes we observe in adult KO mice represent developmental deficits, compensatory responses, or ongoing regulatory abnormalities.

In terms of mechanism, several questions remain. It is yet to be determined 1) whether differences in dendritic morphology in MD-M are due to FMRP’s regulation of mRNAs or its direct protein–protein interactions (Deng et al. 2013), 2) if the dendritic changes identified here are primary or compensatory (Portera-Cailliau 2012), and 3) whether acute treatment would restore dendritic phenotypes in the adult brain (Restivo et al. 2005; Dolan et al. 2013).

FXS is characterized by disabilities in both the cognitive and social/emotional domains. Our studies of MD in *Fmr1* KO mice reveal changes in both the cognitive and social/emotional MD circuits: physiological in MD-L and morphological in MD-M. As with previous studies, changes after *Fmr1* KO are cell-type dependent and importantly reveal the independence of physiological and morphological features. In total, our findings offer new insights into how this complex gene regulates different facets of neuronal structure and function, potentially contributing to the cognitive and affective symptoms of FXS.

## Supplementary Information

Below is the link to the electronic supplementary material.

Supplementary file1 (HTML 7487 kb)

Supplemental Figure 1. Complete dataset of reconstructed MD→mPFC neurons organized by anatomical position, subnucleus, genotype, and complexity of dendritic arbor. All dendritic reconstructions from wildtype (WT) and Fmr1 knockout (KO) mice are shown organized by anterior-posterior position (rows, indicated in mm posterior to Bregma), subnucleus (MD-M in dark blue panels, MD-L in light blue panels), and genotype (separated by dashed horizontal lines within each panel). Left column shows representative CTB fluorescence images from Figure 1 with medial (M) and lateral (L) subnuclei outlined, corresponding to each anterior-posterior level. Scale bars in fluorescence images: 200 μm. Each neuronal reconstruction represents one recorded neuron that was filled with biocytin, fixed, processed, and traced using Neurolucida. Neurons are arranged from anterior (top, -0.94 mm) to posterior (bottom, -1.46 mm). Gradient bars illustrate how neurons are ordered from less complex (left) to more complex (right). Scale bar for all reconstructions: 200 μm. Black dots represent cut processes. Axons shown in red. Quantitative analyses of these neurons are presented in Figures 2-5 and Tables 1-3

Supplemental Figure 2. Map of approximate locations of reconstructed neurons with estimated distance from Bregma (in mm). Black dots represent cut processes. Axons shown in red

Supplemental Figure 3. Quantifications of dendritic morphology. Illustrations of dendrite measurements in this report. 2D Sholl analysis measures dendritic length and total number of intersections in each concentric circle. Primary dendrites are the branches originating from the cell body. Area and volume estimate the overall size of the dendritic arbor. Nodes are the points at which branches divide. Terminals are the natural ending of a branch. Dendritic complexity is a function of the “branchiness” of the dendritic arbor

Supplementary file5 (XLSX 15 kb)

## Data Availability

Data that support the findings of the study are deposited in https://github.com/BrumbackLab/MD-M_and_MD-L-dendritic_morphology_in_Fmr1-WT_and_KO_mice

## Abbreviations

MD: Mediodorsal thalamus
mPFC: Medial prefrontal cortex
MD-M: Medial subnucleus of the mediodorsal thalamus
MD-L: Lateral subnucleus of the mediodorsal thalamus
*Fmr1*: Gene causing fragile X syndrome and related conditions
FMRP: The protein encoded by *Fmr1*
CTB: Cholera toxin, subunit B
WT: Wildtype
KO: Knockout
