# Supplementary material for: Loss of Fmr1 causes subnucleus-specific decreases in dendritic complexity in mediodorsal thalamus
Source: Brain Struct Funct. 2026 Oct 14;231(8):152. doi: 10.1007/s00429-026-03179-5 (PMC13669028; doi:10.1007/s00429-026-03179-5)
Supplement: Supplementary file 1 [file 429_2026_3179_MOESM1_ESM.html]

FMR1 Morphology Analysis: Alternative Statistical Approaches


Code 

- Show All Code
- Hide All Code

# FMR1 Morphology Analysis: Alternative Statistical Approaches

#### Sensitivity Analysis for Kizimenko, Lyuboslavsky, & Brumback

#### 2025-09-05

## Introduction

This analysis explores alternative statistical approaches to detect
potential biological effects of Fmr1 knockout on dendritic morphology in
the mediodorsal thalamus (MD) of mice. The focus is on applying
different analytical methods that will be sensitive to real biological
differences with scientific rigor.

## Libraries and Data Preparation

```
# Load required libraries
library(lme4)        # For linear mixed effects models
library(lmerTest)    # For p-values with lmer models
library(ggplot2)     # For visualization
library(dplyr)       # For data manipulation
library(tidyr)       # For data reshaping
library(emmeans)     # For post-hoc comparisons
library(broom.mixed) # For tidy extraction of model results
library(stats)       # For basic statistical tests
library(coin)        # For permutation tests
library(boot)        # For bootstrapping
library(rcompanion)  # For additional statistical tools
library(effsize)     # For effect size calculations
```

```
# Read in the morphology data
morphology_data <- read.csv("FMR1_Morphology_Data_for_R_Analysis.csv", stringsAsFactors = FALSE)

# Data preparation: Convert data types and create proper identifiers
morphology_data <- morphology_data %>%
  mutate(
    # Convert Genotype from numeric to factor (0 = KO, 1 = WT)
    Genotype = factor(ifelse(Genotype == 1, "WT", "KO"), 
                      levels = c("WT", "KO")),
    
    # Convert Subnucleus from numeric to factor (0 = M, 2 = L)
    Region = factor(ifelse(Subnucleus == 0, "M", "L"), 
                    levels = c("M", "L")),
    
    # Create proper identifiers for the hierarchical structure
    Mouse_ID = factor(Mouse_ID),
    Neuron_ID = factor(Neuron_ID)
  ) %>%
  filter(!is.na(Genotype) & !is.na(Region))  # Remove any rows with missing key data

# Print basic summary of the data
cat("Data summary:\n")
```

```
## Data summary:
```

```
cat("Total number of observations:", nrow(morphology_data), "\n")
```

```
## Total number of observations: 103
```

```
cat("Number of unique mice:", length(unique(morphology_data$Mouse_ID)), "\n")
```

```
## Number of unique mice: 50
```

```
cat("Number of unique neurons:", length(unique(morphology_data$Neuron_ID)), "\n\n")
```

```
## Number of unique neurons: 103
```

```
cat("Genotype distribution:\n")
```

```
## Genotype distribution:
```

```
print(table(morphology_data$Genotype))
```

```
## 
## WT KO 
## 50 53
```

```
cat("\nRegion distribution:\n")
```

```
## 
## Region distribution:
```

```
print(table(morphology_data$Region))
```

```
## 
##  M  L 
## 52 51
```

```
cat("\nGenotype x Region distribution:\n")
```

```
## 
## Genotype x Region distribution:
```

```
print(table(morphology_data$Genotype, morphology_data$Region))
```

```
##     
##       M  L
##   WT 26 24
##   KO 26 27
```

```
# Check the number of neurons per mouse to ensure sufficient replication
neurons_per_mouse <- morphology_data %>%
  group_by(Mouse_ID, Genotype, Region) %>%
  summarize(Neurons = n(), .groups = "drop")

cat("\nNeurons per mouse by genotype and region:\n")
```

```
## 
## Neurons per mouse by genotype and region:
```

```
print(neurons_per_mouse)
```

```
## # A tibble: 69 × 4
##    Mouse_ID Genotype Region Neurons
##    <fct>    <fct>    <fct>    <int>
##  1 190813   WT       L            1
##  2 190924   WT       M            1
##  3 190925   WT       L            1
##  4 191015   KO       L            1
##  5 191030   KO       L            3
##  6 200323   WT       M            1
##  7 200324   WT       M            1
##  8 200901   WT       M            1
##  9 200901   WT       L            2
## 10 200922   WT       M            1
## # ℹ 59 more rows
```

## Descriptive Statistics

```
# Define key measures to analyze
measures <- c("total_dendritic_length_in_um", "DendriticComplexity", 
              "Number_of_primary_dendrites", "NodesCount", "Terminals")

# Create descriptive statistics
descriptive_stats <- morphology_data %>%
  group_by(Genotype, Region) %>%
  summarize(across(all_of(measures),
                   list(
                     mean = ~mean(., na.rm = TRUE),
                     median = ~median(., na.rm = TRUE),
                     sd = ~sd(., na.rm = TRUE),
                     se = ~sd(., na.rm = TRUE) / sqrt(n()),
                     n = ~n()
                   )),
            .groups = "drop")

# Display the statistics in a more readable format for MD-M region
knitr::kable(
  descriptive_stats %>%
    filter(Region == "M") %>%
    select(Genotype, 
           contains("total_dendritic_length"), 
           contains("DendriticComplexity"),
           contains("NodesCount")),
  caption = "Descriptive statistics for key morphological measures in MD-M region"
)
```

Descriptive statistics for key morphological measures in MD-M
region

| Genotype | total\_dendritic\_length\_in\_um\_mean | total\_dendritic\_length\_in\_um\_median | total\_dendritic\_length\_in\_um\_sd | total\_dendritic\_length\_in\_um\_se | total\_dendritic\_length\_in\_um\_n | DendriticComplexity\_mean | DendriticComplexity\_median | DendriticComplexity\_sd | DendriticComplexity\_se | DendriticComplexity\_n | NodesCount\_mean | NodesCount\_median | NodesCount\_sd | NodesCount\_se | NodesCount\_n |
| --- | --- | --- | --- | --- | --- | --- | --- | --- | --- | --- | --- | --- | --- | --- | --- |
| WT | 5770.777 | 5793.76 | 1524.186 | 298.9174 | 26 | 332288.7 | 287125 | 189348.4 | 37134.27 | 26 | 44.19231 | 41.5 | 12.06655 | 2.366444 | 26 |
| KO | 4575.582 | 4434.72 | 1658.883 | 325.3338 | 26 | 222864.5 | 176549 | 155360.9 | 30468.78 | 26 | 34.65385 | 31.0 | 12.89943 | 2.529787 | 26 |

```
# Display the statistics in a more readable format for MD-L region
knitr::kable(
  descriptive_stats %>%
    filter(Region == "L") %>%
    select(Genotype, 
           contains("total_dendritic_length"), 
           contains("DendriticComplexity"),
           contains("NodesCount")),
  caption = "Descriptive statistics for key morphological measures in MD-L region"
)
```

Descriptive statistics for key morphological measures in MD-L
region

| Genotype | total\_dendritic\_length\_in\_um\_mean | total\_dendritic\_length\_in\_um\_median | total\_dendritic\_length\_in\_um\_sd | total\_dendritic\_length\_in\_um\_se | total\_dendritic\_length\_in\_um\_n | DendriticComplexity\_mean | DendriticComplexity\_median | DendriticComplexity\_sd | DendriticComplexity\_se | DendriticComplexity\_n | NodesCount\_mean | NodesCount\_median | NodesCount\_sd | NodesCount\_se | NodesCount\_n |
| --- | --- | --- | --- | --- | --- | --- | --- | --- | --- | --- | --- | --- | --- | --- | --- |
| WT | 5406.517 | 5222.545 | 2000.844 | 408.4206 | 24 | 318534.3 | 267046.5 | 227974.7 | 46535.15 | 24 | 43.95833 | 44.5 | 16.94102 | 3.458071 | 24 |
| KO | 5515.667 | 5250.170 | 1597.183 | 307.3780 | 27 | 315380.6 | 284645.0 | 169587.2 | 32637.06 | 27 | 45.70370 | 45.0 | 13.65457 | 2.627823 | 27 |

```
# Calculate and display percent differences between WT and KO for each region
pct_diff <- descriptive_stats %>%
  select(Genotype, Region, ends_with("_mean")) %>%
  pivot_wider(names_from = Genotype, values_from = ends_with("_mean")) %>%
  mutate(across(ends_with("_WT"), ~ (. - get(gsub("_WT", "_KO", cur_column()))) / . * 100,
                .names = "{.col}_pct_diff"))

knitr::kable(
  pct_diff %>%
    select(Region, ends_with("pct_diff")),
  caption = "Percent difference ((WT-KO)/WT*100) for each measure by region"
)
```

Percent difference ((WT-KO)/WT\*100) for each measure by
region

| Region | total\_dendritic\_length\_in\_um\_mean\_WT\_pct\_diff | DendriticComplexity\_mean\_WT\_pct\_diff | Number\_of\_primary\_dendrites\_mean\_WT\_pct\_diff | NodesCount\_mean\_WT\_pct\_diff | Terminals\_mean\_WT\_pct\_diff |
| --- | --- | --- | --- | --- | --- |
| M | 20.711157 | 32.9304492 | 6.766917 | 21.583986 | 20.549020 |
| L | -2.018858 | 0.9900685 | -5.461394 | -3.970511 | -4.710041 |

## Approach 1: Simple t-tests

```
# Function to run t-tests for a specific measure and region
run_t_test <- function(data, measure, region) {
  # Filter data for the specific region
  region_data <- data %>% filter(Region == region)
  
  # Run two-tailed t-test
  t_result <- t.test(
    region_data[[measure]] ~ region_data$Genotype,
    var.equal = FALSE  # Welch's t-test (doesn't assume equal variances)
  )
  
  # Run Wilcoxon rank-sum test (non-parametric)
  w_result <- wilcox.test(
    region_data[[measure]] ~ region_data$Genotype,
    exact = FALSE,
    correct = TRUE
  )
  
  # Calculate Cohen's d effect size
  d_result <- cohen.d(
    region_data[[measure]] ~ region_data$Genotype,
    na.rm = TRUE
  )
  
  # Return results
  return(list(
    measure = measure,
    region = region,
    t_test = t_result,
    wilcox_test = w_result,
    cohens_d = d_result
  ))
}

# Run t-tests for all measures in MD-M region
mdm_t_tests <- lapply(measures, function(m) run_t_test(morphology_data, m, "M"))
names(mdm_t_tests) <- measures

# Run t-tests for all measures in MD-L region
mdl_t_tests <- lapply(measures, function(m) run_t_test(morphology_data, m, "L"))
names(mdl_t_tests) <- measures

# Function to extract and format results
extract_t_test_results <- function(t_test_list, region) {
  # Initialize data frame to store results
  results <- data.frame(
    Measure = character(),
    Region = character(),
    T_value = numeric(),
    Two_tailed_p = numeric(),
    Wilcoxon_p = numeric(),
    Cohens_d = numeric(),
    Effect_size = character(),
    stringsAsFactors = FALSE
  )
  
  for (m in names(t_test_list)) {
    # Format measure name for display
    measure_name <- gsub("_", " ", m)
    measure_name <- gsub("in um", "(µm)", measure_name)
    measure_name <- tools::toTitleCase(measure_name)
    
    # Extract results
    t_result <- t_test_list[[m]]$t_test
    w_result <- t_test_list[[m]]$wilcox_test
    d_value <- t_test_list[[m]]$cohens_d$estimate
    
    # Categorize effect size
    effect_size_cat <- ifelse(abs(d_value) < 0.2, "Negligible",
                             ifelse(abs(d_value) < 0.5, "Small",
                                   ifelse(abs(d_value) < 0.8, "Medium", "Large")))
    
    # Add to results
    results <- rbind(results, data.frame(
      Measure = measure_name,
      Region = region,
      T_value = t_result$statistic,
      Two_tailed_p = t_result$p.value,
      Wilcoxon_p = w_result$p.value,
      Cohens_d = d_value,
      Effect_size = effect_size_cat,
      stringsAsFactors = FALSE
    ))
  }
  
  # Sort by p-value
  results <- results[order(results$Two_tailed_p), ]
  
  return(results)
}

# Extract and format results
mdm_t_results <- extract_t_test_results(mdm_t_tests, "MD-M")
mdl_t_results <- extract_t_test_results(mdl_t_tests, "MD-L")

# Add significance indicators
add_sig_indicators <- function(results) {
  results %>%
    mutate(
      Two_tailed_sig = case_when(
        Two_tailed_p < 0.001 ~ "***",
        Two_tailed_p < 0.01 ~ "**",
        Two_tailed_p < 0.05 ~ "*",
        Two_tailed_p < 0.1 ~ ".",
        TRUE ~ "ns"
      ),
      Wilcoxon_sig = case_when(
        Wilcoxon_p < 0.001 ~ "***",
        Wilcoxon_p < 0.01 ~ "**",
        Wilcoxon_p < 0.05 ~ "*",
        Wilcoxon_p < 0.1 ~ ".",
        TRUE ~ "ns"
      )
    )
}

mdm_t_results <- add_sig_indicators(mdm_t_results)
mdl_t_results <- add_sig_indicators(mdl_t_results)

# Format p-values
format_p_values <- function(results) {
  results %>%
    mutate(
      Two_tailed_p_fmt = ifelse(Two_tailed_p < 0.001, 
                              "<0.001", 
                              sprintf("%.4f", Two_tailed_p)),
      Wilcoxon_p_fmt = ifelse(Wilcoxon_p < 0.001, 
                             "<0.001", 
                             sprintf("%.4f", Wilcoxon_p)),
      Cohens_d_fmt = sprintf("%.2f", Cohens_d)
    )
}

mdm_t_results_fmt <- format_p_values(mdm_t_results)
mdl_t_results_fmt <- format_p_values(mdl_t_results)

# Display results for MD-M region
cat("T-test results for MD-M region:\n")
```

```
## T-test results for MD-M region:
```

```
knitr::kable(
  mdm_t_results_fmt %>%
    select(Measure, T_value, 
           Two_tailed_p_fmt, Two_tailed_sig,
           Wilcoxon_p_fmt, Wilcoxon_sig,
           Cohens_d_fmt, Effect_size),
  col.names = c("Measure", "t-value", 
                "Two-tailed p", "Sig.", 
                "Wilcoxon p", "Sig.",
                "Cohen's d", "Effect Size"),
  caption = "t-test, Wilcoxon test, and effect size results for MD-M region"
)
```

t-test, Wilcoxon test, and effect size results for MD-M
region

|  | Measure | t-value | Two-tailed p | Sig. | Wilcoxon p | Sig. | Cohen’s d | Effect Size |
| --- | --- | --- | --- | --- | --- | --- | --- | --- |
| t4 | Terminals | 2.9030226 | 0.0055 | \*\* | 0.0059 | \*\* | 0.81 | Large |
| t3 | NodesCount | 2.7535302 | 0.0082 | \*\* | 0.0088 | \*\* | 0.76 | Medium |
| t | Total Dendritic Length (Μm) | 2.7052397 | 0.0093 | \*\* | 0.0113 | \* | 0.75 | Medium |
| t1 | DendriticComplexity | 2.2780389 | 0.0272 | \* | 0.0345 | \* | 0.63 | Medium |
| t2 | Number of Primary Dendrites | 0.9912279 | 0.3264 | ns | 0.3905 | ns | 0.27 | Small |

```
# Display results for MD-L region
cat("T-test results for MD-L region:\n")
```

```
## T-test results for MD-L region:
```

```
knitr::kable(
  mdl_t_results_fmt %>%
    select(Measure, T_value, 
           Two_tailed_p_fmt, Two_tailed_sig,
           Wilcoxon_p_fmt, Wilcoxon_sig,
           Cohens_d_fmt, Effect_size),
  col.names = c("Measure", "t-value", 
                "Two-tailed p", "Sig.", 
                "Wilcoxon p", "Sig.",
                "Cohen's d", "Effect Size"),
  caption = "t-test, Wilcoxon test, and effect size results for MD-L region"
)
```

t-test, Wilcoxon test, and effect size results for MD-L
region

|  | Measure | t-value | Two-tailed p | Sig. | Wilcoxon p | Sig. | Cohen’s d | Effect Size |
| --- | --- | --- | --- | --- | --- | --- | --- | --- |
| t2 | Number of Primary Dendrites | -0.8405251 | 0.4052 | ns | 0.2677 | ns | -0.24 | Small |
| t4 | Terminals | -0.5127507 | 0.6106 | ns | 0.5773 | ns | -0.15 | Negligible |
| t3 | NodesCount | -0.4018593 | 0.6897 | ns | 0.6710 | ns | -0.11 | Negligible |
| t | Total Dendritic Length (Μm) | -0.2135321 | 0.8319 | ns | 0.8282 | ns | -0.06 | Negligible |
| t1 | DendriticComplexity | 0.0554847 | 0.9560 | ns | 0.6304 | ns | 0.02 | Negligible |

## Approach 2: Mixed Effects Models Analysis

```
# Function to run mixed model for a specific region and measure
run_region_specific_model <- function(data, measure, region) {
  # Filter data for the specific region
  region_data <- data %>% filter(Region == region)
  
  # Define the formula (simpler model without Region interaction)
  formula <- as.formula(paste0(measure, " ~ Genotype + (1|Mouse_ID)"))
  
  # Run the model
  model <- tryCatch({
    lmer(formula, 
         data = region_data,
         control = lmerControl(optimizer = "bobyqa", 
                              optCtrl = list(maxfun = 100000)))
  }, error = function(e) {
    cat("Error in mixed model for measure:", measure, "in region:", region, "\n")
    cat("Error message:", e$message, "\n")
    return(NULL)
  })
  
  # Return the model
  return(model)
}

# Run models separately for each measure and region
cat("Running separate mixed models for each region and measure...\n")
```

```
## Running separate mixed models for each region and measure...
```

```
# Initialize result structures
mdm_mixed_results <- list()
mdl_mixed_results <- list()
mdm_mixed_p <- data.frame(Measure=character(), P_value=numeric(), stringsAsFactors=FALSE)
mdl_mixed_p <- data.frame(Measure=character(), P_value=numeric(), stringsAsFactors=FALSE)

# Run models for each measure and region
for (m in measures) {
  # Format measure name for display
  measure_name <- gsub("_", " ", m)
  measure_name <- gsub("in um", "(µm)", measure_name)
  measure_name <- tools::toTitleCase(measure_name)
  
  # Run model for MD-M region
  mdm_model <- run_region_specific_model(morphology_data, m, "M")
  if (!is.null(mdm_model)) {
    mdm_mixed_results[[m]] <- mdm_model
    
    # Extract fixed effects summary
    mdm_summary <- summary(mdm_model)
    mdm_fixed <- mdm_summary$coefficients
    
    # Extract p-value for Genotype effect (should be in row 2)
    if (nrow(mdm_fixed) >= 2) {
      genotype_p <- mdm_fixed[2, "Pr(>|t|)"]
      mdm_mixed_p <- rbind(mdm_mixed_p, 
                        data.frame(Measure=measure_name, 
                                 P_value=genotype_p,
                                 stringsAsFactors=FALSE))
    }
  }
  
  # Run model for MD-L region
  mdl_model <- run_region_specific_model(morphology_data, m, "L")
  if (!is.null(mdl_model)) {
    mdl_mixed_results[[m]] <- mdl_model
    
    # Extract fixed effects summary
    mdl_summary <- summary(mdl_model)
    mdl_fixed <- mdl_summary$coefficients
    
    # Extract p-value for Genotype effect (should be in row 2)
    if (nrow(mdl_fixed) >= 2) {
      genotype_p <- mdl_fixed[2, "Pr(>|t|)"]
      mdl_mixed_p <- rbind(mdl_mixed_p, 
                        data.frame(Measure=measure_name, 
                                 P_value=genotype_p,
                                 stringsAsFactors=FALSE))
    }
  }
}

# Display the mixed model p-values
cat("MD-M mixed model p-values:\n")
```

```
## MD-M mixed model p-values:
```

```
print(mdm_mixed_p)
```

```
##                       Measure     P_value
## 1 Total Dendritic Length (Μm) 0.009308338
## 2         DendriticComplexity 0.022724261
## 3 Number of Primary Dendrites 0.326347164
## 4                  NodesCount 0.008197173
## 5                   Terminals 0.005486287
```

```
cat("\nMD-L mixed model p-values:\n")
```

```
## 
## MD-L mixed model p-values:
```

```
print(mdl_mixed_p)
```

```
##                       Measure   P_value
## 1 Total Dendritic Length (Μm) 0.6526707
## 2         DendriticComplexity 0.9549825
## 3 Number of Primary Dendrites 0.4239492
## 4                  NodesCount 0.5897537
## 5                   Terminals 0.5115549
```

```
# Create function to nicely format mixed model results for display
format_mixed_model_display <- function(model, measure_name, region_name) {
  if (is.null(model)) {
    cat(paste0("\n### ", measure_name, " - ", region_name, " Region\n\n"))
    cat("No mixed model results available for this measure and region.\n")
    return()
  }
  
  # Get model summary
  model_summary <- summary(model)
  
  # Extract fixed effects table
  fixed_effects <- as.data.frame(model_summary$coefficients)
  
  # Format the table
  fixed_effects$p_formatted <- ifelse(fixed_effects[, "Pr(>|t|)"] < 0.001, 
                                   "<0.001", 
                                   sprintf("%.4f", fixed_effects[, "Pr(>|t|)"]))
  
  # Add significance indicators
  fixed_effects$Significance <- case_when(
    fixed_effects[, "Pr(>|t|)"] < 0.001 ~ "***",
    fixed_effects[, "Pr(>|t|)"] < 0.01 ~ "**",
    fixed_effects[, "Pr(>|t|)"] < 0.05 ~ "*",
    fixed_effects[, "Pr(>|t|)"] < 0.1 ~ ".",
    TRUE ~ "ns"
  )
  
  # Print header
  cat(paste0("\n### ", measure_name, " - ", region_name, " Region\n\n"))
  
  # Print fixed effects table
  knitr::kable(
    fixed_effects %>%
      select("Estimate", "Std. Error", "df", "t value", "p_formatted", "Significance"),
    col.names = c("Estimate", "Std. Error", "df", "t-value", "p-value", "Sig."),
    caption = paste0("Mixed model results for ", measure_name, " in ", region_name, " region")
  )
  
  # Add some model fit statistics
  cat("\nModel fit statistics:\n")
  cat("AIC:", AIC(model), "\n")
  cat("BIC:", BIC(model), "\n")
  
  # Add random effects variance
  cat("\nRandom effects variance:\n")
  print(VarCorr(model))
}

# Display results for each measure in MD-M region
cat("## Mixed Model Results - MD-M Region\n\n")
```

```
## ## Mixed Model Results - MD-M Region
```

```
for (m in names(mdm_mixed_results)) {
  # Format measure name for display
  measure_name <- gsub("_", " ", m)
  measure_name <- gsub("in um", "(µm)", measure_name)
  measure_name <- tools::toTitleCase(measure_name)
  
  format_mixed_model_display(mdm_mixed_results[[m]], measure_name, "MD-M")
}
```

```
## 
## ### Total Dendritic Length (Μm) - MD-M Region
## 
## 
## Model fit statistics:
## AIC: 893.7449 
## BIC: 901.5499 
## 
## Random effects variance:
##  Groups   Name        Std.Dev.  
##  Mouse_ID (Intercept) 2.1567e-04
##  Residual             1.5930e+03
## 
## ### DendriticComplexity - MD-M Region
## 
## 
## Model fit statistics:
## AIC: 1362.625 
## BIC: 1370.43 
## 
## Random effects variance:
##  Groups   Name        Std.Dev.
##  Mouse_ID (Intercept)      0  
##  Residual             173190  
## 
## ### Number of Primary Dendrites - MD-M Region
## 
## 
## Model fit statistics:
## AIC: 179.4514 
## BIC: 187.2564 
## 
## Random effects variance:
##  Groups   Name        Std.Dev.
##  Mouse_ID (Intercept) 0.0000  
##  Residual             1.2591  
## 
## ### NodesCount - MD-M Region
## 
## 
## Model fit statistics:
## AIC: 408.9024 
## BIC: 416.7073 
## 
## Random effects variance:
##  Groups   Name        Std.Dev.  
##  Mouse_ID (Intercept) 4.6481e-07
##  Residual             1.2490e+01
## 
## ### Terminals - MD-M Region
## 
## 
## Model fit statistics:
## AIC: 409.1071 
## BIC: 416.912 
## 
## Random effects variance:
##  Groups   Name        Std.Dev.
##  Mouse_ID (Intercept)  0.000  
##  Residual             12.516
```

```
# Display results for each measure in MD-L region
cat("\n## Mixed Model Results - MD-L Region\n\n")
```

```
## 
## ## Mixed Model Results - MD-L Region
```

```
for (m in names(mdl_mixed_results)) {
  # Format measure name for display
  measure_name <- gsub("_", " ", m)
  measure_name <- gsub("in um", "(µm)", measure_name)
  measure_name <- tools::toTitleCase(measure_name)
  
  format_mixed_model_display(mdl_mixed_results[[m]], measure_name, "MD-L")
}
```

```
## 
## ### Total Dendritic Length (Μm) - MD-L Region
## 
## 
## Model fit statistics:
## AIC: 886.4459 
## BIC: 894.1732 
## 
## Random effects variance:
##  Groups   Name        Std.Dev.
##  Mouse_ID (Intercept)  924.63 
##  Residual             1545.12 
## 
## ### DendriticComplexity - MD-L Region
## 
## 
## Model fit statistics:
## AIC: 1349.301 
## BIC: 1357.029 
## 
## Random effects variance:
##  Groups   Name        Std.Dev.  
##  Mouse_ID (Intercept) 5.0958e-02
##  Residual             1.9914e+05
## 
## ### Number of Primary Dendrites - MD-L Region
## 
## 
## Model fit statistics:
## AIC: 163.6494 
## BIC: 171.3767 
## 
## Random effects variance:
##  Groups   Name        Std.Dev.
##  Mouse_ID (Intercept) 0.57285 
##  Residual             0.97087 
## 
## ### NodesCount - MD-L Region
## 
## 
## Model fit statistics:
## AIC: 420.0673 
## BIC: 427.7946 
## 
## Random effects variance:
##  Groups   Name        Std.Dev.
##  Mouse_ID (Intercept)  6.5129 
##  Residual             13.8586 
## 
## ### Terminals - MD-L Region
## 
## 
## Model fit statistics:
## AIC: 422.2869 
## BIC: 430.0142 
## 
## Random effects variance:
##  Groups   Name        Std.Dev.
##  Mouse_ID (Intercept)  7.2104 
##  Residual             13.9522
```

## Approach 3: Bootstrap Analysis

```
# Function to calculate mean difference between WT and KO for bootstrap
mean_diff <- function(data, indices, measure) {
  d <- data[indices, ]
  wt_mean <- mean(d[d$Genotype == "WT", measure], na.rm = TRUE)
  ko_mean <- mean(d[d$Genotype == "KO", measure], na.rm = TRUE)
  return(wt_mean - ko_mean)  # Return the actual difference (not absolute)
}

# Function to run bootstrap analysis for a specific measure and region
run_bootstrap <- function(data, measure, region, R = 2000) {
  # Filter data for the specific region
  region_data <- data %>% filter(Region == region)
  
  # Set up bootstrap
  boot_result <- boot(
    data = region_data,
    statistic = mean_diff,
    R = R,
    measure = measure
  )
  
  # Calculate observed value (WT minus KO)
  obs_wt <- mean(region_data[region_data$Genotype == "WT", measure], na.rm = TRUE)
  obs_ko <- mean(region_data[region_data$Genotype == "KO", measure], na.rm = TRUE)
  obs_diff <- obs_wt - obs_ko
  
  # Calculate two-sided bootstrap p-value properly - FIX #1
  # Test against null hypothesis (difference = 0) rather than against observed difference
  # For two-sided test: 2 * min(p(t <= 0), p(t >= 0))
  boot_p <- 2 * min(mean(boot_result$t <= 0), mean(boot_result$t >= 0))
  
  # Calculate 95% confidence interval
  boot_ci <- try(boot.ci(boot_result, type = "perc"), silent = TRUE)
  
  # Return results
  return(list(
    measure = measure,
    region = region,
    boot_result = boot_result,
    obs_diff = obs_diff,
    boot_p = boot_p,
    boot_ci = if(inherits(boot_ci, "try-error")) NULL else boot_ci
  ))
}

# Run bootstrap analysis for all measures in MD-M region
set.seed(123)  # For reproducibility
mdm_boot <- lapply(measures, function(m) {
  tryCatch(
    run_bootstrap(morphology_data, m, "M"),
    error = function(e) {
      message("Error in bootstrap for measure ", m, ": ", e$message)
      return(NULL)
    }
  )
})
names(mdm_boot) <- measures

# Run bootstrap analysis for all measures in MD-L region
set.seed(123)  # For reproducibility
mdl_boot <- lapply(measures, function(m) {
  tryCatch(
    run_bootstrap(morphology_data, m, "L"),
    error = function(e) {
      message("Error in bootstrap for measure ", m, ": ", e$message)
      return(NULL)
    }
  )
})
names(mdl_boot) <- measures

# Function to extract and format bootstrap results
extract_bootstrap_results <- function(boot_list, region) {
  # Initialize data frame to store results
  results <- data.frame(
    Measure = character(),
    Region = character(),
    Original_diff = numeric(),
    Bootstrap_mean = numeric(),
    Bootstrap_p = numeric(),
    CI_lower = numeric(),
    CI_upper = numeric(),
    stringsAsFactors = FALSE
  )
  
  for (m in names(boot_list)) {
    if (is.null(boot_list[[m]])) next
    
    # Format measure name for display
    measure_name <- gsub("_", " ", m)
    measure_name <- gsub("in um", "(µm)", measure_name)
    measure_name <- tools::toTitleCase(measure_name)
    
    # Extract results
    boot_result <- boot_list[[m]]$boot_result
    obs_diff <- boot_list[[m]]$obs_diff
    boot_p <- boot_list[[m]]$boot_p
    boot_ci <- boot_list[[m]]$boot_ci
    
    # Handle potential NULL boot_ci
    ci_lower <- ci_upper <- NA
    if (!is.null(boot_ci) && !inherits(boot_ci, "try-error")) {
      ci_lower <- boot_ci$percent[4]
      ci_upper <- boot_ci$percent[5]
    }
    
    # Add to results
    results <- rbind(results, data.frame(
      Measure = measure_name,
      Region = region,
      Original_diff = obs_diff,
      Bootstrap_mean = mean(boot_result$t),
      Bootstrap_p = boot_p,
      CI_lower = ci_lower,
      CI_upper = ci_upper,
      stringsAsFactors = FALSE
    ))
  }
  
  # Sort by bootstrap p-value
  results <- results[order(results$Bootstrap_p), ]
  
  return(results)
}

# Extract and format bootstrap results
mdm_boot_results <- extract_bootstrap_results(mdm_boot, "MD-M")
mdl_boot_results <- extract_bootstrap_results(mdl_boot, "MD-L")

# Add significance indicators
add_boot_sig <- function(results) {
  results %>%
    mutate(
      Bootstrap_sig = case_when(
        Bootstrap_p < 0.001 ~ "***",
        Bootstrap_p < 0.01 ~ "**",
        Bootstrap_p < 0.05 ~ "*",
        Bootstrap_p < 0.1 ~ ".",
        TRUE ~ "ns"
      ),
      # FIX #2: Properly interpret CI containing zero
      CI_contains_zero = ifelse((is.na(CI_lower) | is.na(CI_upper)), NA, 
                                ifelse(CI_lower <= 0 & CI_upper >= 0, TRUE, FALSE))
    )
}

mdm_boot_results <- add_boot_sig(mdm_boot_results)
mdl_boot_results <- add_boot_sig(mdl_boot_results)

# Format bootstrap results
format_boot_results <- function(results) {
  results %>%
    mutate(
      Original_diff_fmt = sprintf("%.2f", Original_diff),
      Bootstrap_mean_fmt = sprintf("%.2f", Bootstrap_mean),
      Bootstrap_p_fmt = ifelse(Bootstrap_p < 0.001, 
                             "<0.001", 
                             sprintf("%.4f", Bootstrap_p)),
      CI_fmt = ifelse(is.na(CI_lower) | is.na(CI_upper), 
                     "N/A", 
                     sprintf("%.2f to %.2f", CI_lower, CI_upper))
    )
}

mdm_boot_fmt <- format_boot_results(mdm_boot_results)
mdl_boot_fmt <- format_boot_results(mdl_boot_results)

# Display results for MD-M region
cat("Bootstrap analysis results for MD-M region:\n")
```

```
## Bootstrap analysis results for MD-M region:
```

```
knitr::kable(
  mdm_boot_fmt %>%
    select(Measure, Original_diff_fmt, Bootstrap_mean_fmt, 
           Bootstrap_p_fmt, Bootstrap_sig, CI_fmt, CI_contains_zero),
  col.names = c("Measure", "Original Diff (WT-KO)", "Bootstrap Mean", 
                "Bootstrap p", "Sig.", "95% CI", "CI Contains Zero"),
  caption = "Bootstrap analysis results for MD-M region"
)
```

Bootstrap analysis results for MD-M region

|  | Measure | Original Diff (WT-KO) | Bootstrap Mean | Bootstrap p | Sig. | 95% CI | CI Contains Zero |
| --- | --- | --- | --- | --- | --- | --- | --- |
| 5 | Terminals | 10.08 | 10.13 | 0.0050 | \*\* | 3.25 to 17.32 | FALSE |
| 1 | Total Dendritic Length (Μm) | 1195.19 | 1215.59 | 0.0060 | \*\* | 350.55 to 2053.07 | FALSE |
| 4 | NodesCount | 9.54 | 9.53 | 0.0070 | \*\* | 2.87 to 16.14 | FALSE |
| 2 | DendriticComplexity | 109424.15 | 109760.17 | 0.0210 | \* | 15713.00 to 203259.60 | FALSE |
| 3 | Number of Primary Dendrites | 0.35 | 0.35 | 0.3100 | ns | -0.28 to 1.04 | TRUE |

```
# Display results for MD-L region
cat("Bootstrap analysis results for MD-L region:\n")
```

```
## Bootstrap analysis results for MD-L region:
```

```
knitr::kable(
  mdl_boot_fmt %>%
    select(Measure, Original_diff_fmt, Bootstrap_mean_fmt,
           Bootstrap_p_fmt, Bootstrap_sig, CI_fmt, CI_contains_zero),
  col.names = c("Measure", "Original Diff (WT-KO)", "Bootstrap Mean", 
                "Bootstrap p", "Sig.", "95% CI", "CI Contains Zero"),
  caption = "Bootstrap analysis results for MD-L region"
)
```

Bootstrap analysis results for MD-L region

|  | Measure | Original Diff (WT-KO) | Bootstrap Mean | Bootstrap p | Sig. | 95% CI | CI Contains Zero |
| --- | --- | --- | --- | --- | --- | --- | --- |
| 3 | Number of Primary Dendrites | -0.27 | -0.26 | 0.4250 | ns | -0.86 to 0.36 | TRUE |
| 5 | Terminals | -2.28 | -2.32 | 0.6180 | ns | -10.96 to 6.39 | TRUE |
| 4 | NodesCount | -1.75 | -1.69 | 0.7130 | ns | -9.88 to 6.66 | TRUE |
| 1 | Total Dendritic Length (Μm) | -109.15 | -111.76 | 0.8090 | ns | -1055.80 to 920.68 | TRUE |
| 2 | DendriticComplexity | 3153.71 | 2344.62 | 0.9680 | ns | -110610.68 to 114090.68 | TRUE |

```
# Add visualizations of bootstrap distributions for key measures
cat("\n## Bootstrap Distribution Visualizations\n\n")
```

```
## 
## ## Bootstrap Distribution Visualizations
```

```
# Function to plot bootstrap distribution
plot_bootstrap_dist <- function(boot_result, measure_name, region, observed_diff) {
  # Create a data frame for ggplot
  boot_df <- data.frame(diff = boot_result$t)
  
  # Create the plot
  p <- ggplot(boot_df, aes(x = diff)) +
    geom_histogram(bins = 30, fill = "skyblue", color = "black", alpha = 0.7) +
    geom_vline(xintercept = observed_diff, color = "red", linetype = "dashed", size = 1) +
    geom_vline(xintercept = 0, color = "blue", linetype = "solid", size = 0.5) +
    labs(
      title = paste(measure_name, "in", region, "Region"),
      subtitle = paste("Red dashed line: Observed diff =", round(observed_diff, 2), 
                       ", Blue solid line: Zero difference"),
      x = "WT-KO Difference",
      y = "Frequency"
    ) +
    theme_classic()
  
  return(p)
}

# Plot the most significant measures for MD-M region
if (length(mdm_boot) > 0) {
  # Find the most significant measure in MD-M
  top_measure_mdm <- mdm_boot_results$Measure[1]
  top_measure_idx <- which(names(mdm_boot) == gsub(" ", "_", gsub("\\(µm\\)", "in_um", tolower(top_measure_mdm))))
  
  if (length(top_measure_idx) > 0) {
    boot_result <- mdm_boot[[top_measure_idx]]
    p <- plot_bootstrap_dist(
      boot_result$boot_result, 
      top_measure_mdm,
      "MD-M",
      boot_result$obs_diff
    )
    print(p)
  }
}

# Plot the most significant measures for MD-L region
if (length(mdl_boot) > 0) {
  # Find the most significant measure in MD-L
  top_measure_mdl <- mdl_boot_results$Measure[1]
  top_measure_idx <- which(names(mdl_boot) == gsub(" ", "_", gsub("\\(µm\\)", "in_um", tolower(top_measure_mdl))))
  
  if (length(top_measure_idx) > 0) {
    boot_result <- mdl_boot[[top_measure_idx]]
    p <- plot_bootstrap_dist(
      boot_result$boot_result, 
      top_measure_mdl,
      "MD-L",
      boot_result$obs_diff
    )
    print(p)
  }
}
```

## Approach 4: Permutation Tests

```
# Function to run permutation test for a specific measure and region
run_permutation_test <- function(data, measure, region, nPermutations = 10000) {
  # Filter data for the specific region
  region_data <- data %>% filter(Region == region)
  
  # Convert the measure to a formula for independence_test
  formula <- as.formula(paste(measure, "~ Genotype"))
  
  # Run permutation test
  perm_test <- independence_test(
    formula,
    data = region_data,
    distribution = approximate(nresample = nPermutations)
  )
  
  # Extract p-value
  perm_p <- pvalue(perm_test)
  
  # Return results
  return(list(
    measure = measure,
    region = region,
    perm_test = perm_test,
    perm_p = perm_p
  ))
}

# Run permutation tests for all measures in MD-M region
set.seed(123)  # For reproducibility
mdm_perm <- lapply(measures, function(m) run_permutation_test(morphology_data, m, "M"))
names(mdm_perm) <- measures

# Run permutation tests for all measures in MD-L region
set.seed(123)  # For reproducibility
mdl_perm <- lapply(measures, function(m) run_permutation_test(morphology_data, m, "L"))
names(mdl_perm) <- measures

# Function to extract and format permutation test results
extract_permutation_results <- function(perm_list, region) {
  # Initialize data frame to store results
  results <- data.frame(
    Measure = character(),
    Region = character(),
    Test_statistic = numeric(),
    Permutation_p = numeric(),
    stringsAsFactors = FALSE
  )
  
  for (m in names(perm_list)) {
    # Format measure name for display
    measure_name <- gsub("_", " ", m)
    measure_name <- gsub("in um", "(µm)", measure_name)
    measure_name <- tools::toTitleCase(measure_name)
    
    # Extract results
    perm_test <- perm_list[[m]]$perm_test
    perm_p <- perm_list[[m]]$perm_p
    
    # Add to results
    results <- rbind(results, data.frame(
      Measure = measure_name,
      Region = region,
      Test_statistic = statistic(perm_test),
      Permutation_p = perm_p,
      stringsAsFactors = FALSE
    ))
  }
  
  # Sort by permutation p-value
  results <- results[order(results$Permutation_p), ]
  
  return(results)
}

# Extract and format permutation test results
mdm_perm_results <- extract_permutation_results(mdm_perm, "MD-M")
mdl_perm_results <- extract_permutation_results(mdl_perm, "MD-L")

# Add significance indicators
add_perm_sig <- function(results) {
  results %>%
    mutate(
      Permutation_sig = case_when(
        Permutation_p < 0.001 ~ "***",
        Permutation_p < 0.01 ~ "**",
        Permutation_p < 0.05 ~ "*",
        Permutation_p < 0.1 ~ ".",
        TRUE ~ "ns"
      )
    )
}

mdm_perm_results <- add_perm_sig(mdm_perm_results)
mdl_perm_results <- add_perm_sig(mdl_perm_results)

# Format permutation test results
format_perm_results <- function(results) {
  results %>%
    mutate(
      Test_statistic_fmt = sprintf("%.2f", Test_statistic),
      Permutation_p_fmt = ifelse(Permutation_p < 0.001, 
                               "<0.001", 
                               sprintf("%.4f", Permutation_p))
    )
}

mdm_perm_fmt <- format_perm_results(mdm_perm_results)
mdl_perm_fmt <- format_perm_results(mdl_perm_results)

# Display results for MD-M region
cat("Permutation test results for MD-M region:\n")
```

```
## Permutation test results for MD-M region:
```

```
knitr::kable(
  mdm_perm_fmt %>%
    select(Measure, Test_statistic_fmt, Permutation_p_fmt, Permutation_sig),
  col.names = c("Measure", "Test Statistic", "Permutation p", "Sig."),
  caption = "Permutation test results for MD-M region"
)
```

Permutation test results for MD-M region

|  | Measure | Test Statistic | Permutation p | Sig. |
| --- | --- | --- | --- | --- |
| 5 | Terminals | 2.71 | 0.0058 | \*\* |
| 4 | NodesCount | 2.59 | 0.0083 | \*\* |
| 1 | Total Dendritic Length (Μm) | 2.55 | 0.0085 | \*\* |
| 2 | DendriticComplexity | 2.19 | 0.0270 | \* |
| 3 | Number of Primary Dendrites | 0.99 | 0.3775 | ns |

```
# Display results for MD-L region
cat("Permutation test results for MD-L region:\n")
```

```
## Permutation test results for MD-L region:
```

```
knitr::kable(
  mdl_perm_fmt %>%
    select(Measure, Test_statistic_fmt, Permutation_p_fmt, Permutation_sig),
  col.names = c("Measure", "Test Statistic", "Permutation p", "Sig."),
  caption = "Permutation test results for MD-L region"
)
```

Permutation test results for MD-L region

|  | Measure | Test Statistic | Permutation p | Sig. |
| --- | --- | --- | --- | --- |
| 3 | Number of Primary Dendrites | -0.85 | 0.4585 | ns |
| 5 | Terminals | -0.52 | 0.6064 | ns |
| 4 | NodesCount | -0.41 | 0.6882 | ns |
| 1 | Total Dendritic Length (Μm) | -0.22 | 0.8318 | ns |
| 2 | DendriticComplexity | 0.06 | 0.9563 | ns |

## Combined Results Across Approaches

```
# Create combined results dataframes - FIXED!
create_combined_results <- function(t_results, mixed_p, boot_results, perm_results) {
  # Initialize data frame
  combined_results <- data.frame(
    Measure = t_results$Measure,
    
    # T-test results
    T_test_p = t_results$Two_tailed_p,
    T_test_p_fmt = t_results$Two_tailed_p_fmt,
    T_test_p_sig = t_results$Two_tailed_sig,
    
    # Wilcoxon results
    Wilcoxon_p = t_results$Wilcoxon_p,
    Wilcoxon_p_fmt = t_results$Wilcoxon_p_fmt,
    Wilcoxon_p_sig = t_results$Wilcoxon_sig,
    
    # Cohen's d
    Cohens_d = t_results$Cohens_d,
    Cohens_d_fmt = t_results$Cohens_d_fmt,
    Effect_size = t_results$Effect_size,
    
    # Mixed model results
    Mixed_p = NA,
    Mixed_p_fmt = NA,
    Mixed_p_sig = NA,
    
    # Bootstrap results
    Bootstrap_p = NA,
    Bootstrap_p_fmt = NA,
    Bootstrap_p_sig = NA,
    
    # Permutation results
    Permutation_p = NA,
    Permutation_p_fmt = NA,
    Permutation_p_sig = NA,
    
    stringsAsFactors = FALSE
  )
  
  # Add mixed model p-values
  for (i in 1:nrow(combined_results)) {
    measure_name <- combined_results$Measure[i]
    idx <- which(mixed_p$Measure == measure_name)
    if (length(idx) > 0) {
      combined_results$Mixed_p[i] <- mixed_p$P_value[idx]
      combined_results$Mixed_p_fmt[i] <- ifelse(mixed_p$P_value[idx] < 0.001, 
                                          "<0.001", 
                                          sprintf("%.4f", mixed_p$P_value[idx]))
      combined_results$Mixed_p_sig[i] <- case_when(
        mixed_p$P_value[idx] < 0.001 ~ "***",
        mixed_p$P_value[idx] < 0.01 ~ "**",
        mixed_p$P_value[idx] < 0.05 ~ "*",
        mixed_p$P_value[idx] < 0.1 ~ ".",
        TRUE ~ "ns"
      )
    }
  }
  
  # Add bootstrap p-values - FIXED to use proper variable names
  for (i in 1:nrow(combined_results)) {
    measure_name <- combined_results$Measure[i]
    # Find matching measure in bootstrap results
    if (!is.null(boot_results) && nrow(boot_results) > 0) {
      idx <- which(boot_results$Measure == measure_name)
      if (length(idx) > 0) {
        combined_results$Bootstrap_p[i] <- boot_results$Bootstrap_p[idx]
        combined_results$Bootstrap_p_fmt[i] <- boot_results$Bootstrap_p_fmt[idx]
        combined_results$Bootstrap_p_sig[i] <- boot_results$Bootstrap_sig[idx]
      }
    }
  }
  
  # Add permutation p-values
  for (i in 1:nrow(combined_results)) {
    measure_name <- combined_results$Measure[i]
    idx <- which(perm_results$Measure == measure_name)
    if (length(idx) > 0) {
      combined_results$Permutation_p[i] <- perm_results$Permutation_p[idx]
      combined_results$Permutation_p_fmt[i] <- perm_results$Permutation_p_fmt[idx]
      combined_results$Permutation_p_sig[i] <- perm_results$Permutation_sig[idx]
    }
  }
  
  return(combined_results)
}

# Create combined results for MD-M region
mdm_combined_fmt_global <- create_combined_results(
  mdm_t_results_fmt, 
  mdm_mixed_p, 
  mdm_boot_fmt,
  mdm_perm_fmt
)

# Create combined results for MD-L region
mdl_combined_fmt_global <- create_combined_results(
  mdl_t_results_fmt, 
  mdl_mixed_p, 
  mdl_boot_fmt,
  mdl_perm_fmt
)

# Display combined results for MD-M region
cat("Combined results for MD-M region:\n")
```

```
## Combined results for MD-M region:
```

```
knitr::kable(
  mdm_combined_fmt_global %>%
    select(Measure, 
           T_test_p_fmt, T_test_p_sig,
           Wilcoxon_p_fmt, Wilcoxon_p_sig,
           Mixed_p_fmt, Mixed_p_sig,
           Bootstrap_p_fmt, Bootstrap_p_sig,
           Permutation_p_fmt, Permutation_p_sig,
           Cohens_d_fmt, Effect_size),
  col.names = c("Measure", 
                "T-test p", "Sig.", 
                "Wilcoxon p", "Sig.",
                "Mixed p", "Sig.",
                "Bootstrap p", "Sig.",
                "Permutation p", "Sig.",
                "Cohen's d", "Effect Size"),
  caption = "Combined results across all approaches for MD-M region"
)
```

Combined results across all approaches for MD-M
region

| Measure | T-test p | Sig. | Wilcoxon p | Sig. | Mixed p | Sig. | Bootstrap p | Sig. | Permutation p | Sig. | Cohen’s d | Effect Size |
| --- | --- | --- | --- | --- | --- | --- | --- | --- | --- | --- | --- | --- |
| Terminals | 0.0055 | \*\* | 0.0059 | \*\* | 0.0055 | \*\* | 0.0050 | \*\* | 0.0058 | \*\* | 0.81 | Large |
| NodesCount | 0.0082 | \*\* | 0.0088 | \*\* | 0.0082 | \*\* | 0.0070 | \*\* | 0.0083 | \*\* | 0.76 | Medium |
| Total Dendritic Length (Μm) | 0.0093 | \*\* | 0.0113 | \* | 0.0093 | \*\* | 0.0060 | \*\* | 0.0085 | \*\* | 0.75 | Medium |
| DendriticComplexity | 0.0272 | \* | 0.0345 | \* | 0.0227 | \* | 0.0210 | \* | 0.0270 | \* | 0.63 | Medium |
| Number of Primary Dendrites | 0.3264 | ns | 0.3905 | ns | 0.3263 | ns | 0.3100 | ns | 0.3775 | ns | 0.27 | Small |

```
# Display combined results for MD-L region
cat("Combined results for MD-L region:\n")
```

```
## Combined results for MD-L region:
```

```
knitr::kable(
  mdl_combined_fmt_global %>%
    select(Measure, 
           T_test_p_fmt, T_test_p_sig,
           Wilcoxon_p_fmt, Wilcoxon_p_sig,
           Mixed_p_fmt, Mixed_p_sig,
           Bootstrap_p_fmt, Bootstrap_p_sig,
           Permutation_p_fmt, Permutation_p_sig,
           Cohens_d_fmt, Effect_size),
  col.names = c("Measure", 
                "T-test p", "Sig.", 
                "Wilcoxon p", "Sig.",
                "Mixed p", "Sig.",
                "Bootstrap p", "Sig.",
                "Permutation p", "Sig.",
                "Cohen's d", "Effect Size"),
  caption = "Combined results across all approaches for MD-L region"
)
```

Combined results across all approaches for MD-L
region

| Measure | T-test p | Sig. | Wilcoxon p | Sig. | Mixed p | Sig. | Bootstrap p | Sig. | Permutation p | Sig. | Cohen’s d | Effect Size |
| --- | --- | --- | --- | --- | --- | --- | --- | --- | --- | --- | --- | --- |
| Number of Primary Dendrites | 0.4052 | ns | 0.2677 | ns | 0.4239 | ns | 0.4250 | ns | 0.4585 | ns | -0.24 | Small |
| Terminals | 0.6106 | ns | 0.5773 | ns | 0.5116 | ns | 0.6180 | ns | 0.6064 | ns | -0.15 | Negligible |
| NodesCount | 0.6897 | ns | 0.6710 | ns | 0.5898 | ns | 0.7130 | ns | 0.6882 | ns | -0.11 | Negligible |
| Total Dendritic Length (Μm) | 0.8319 | ns | 0.8282 | ns | 0.6527 | ns | 0.8090 | ns | 0.8318 | ns | -0.06 | Negligible |
| DendriticComplexity | 0.9560 | ns | 0.6304 | ns | 0.9550 | ns | 0.9680 | ns | 0.9563 | ns | 0.02 | Negligible |

## Visualizations with Significance Levels

```
# FIXED: Create plots function that correctly references the global variables
create_plots <- function(data, measure, region) {
  # Get the correct combined results based on the region
  combined_results <- if (region == "M") {
    mdm_combined_fmt_global  # Use the global version
  } else {
    mdl_combined_fmt_global  # Use the global version
  }
  
  # Filter data for the specific region
  region_data <- data %>% filter(Region == region)
  
  # Format measure name for display
  measure_clean <- gsub("_", " ", measure)
  measure_clean <- gsub("in um", "(µm)", measure_clean)
  measure_clean <- tools::toTitleCase(measure_clean)
  
  # Get p-values
  row_idx <- which(combined_results$Measure == measure_clean)
  if (length(row_idx) == 0) {
    # Try to match after formatting
    row_idx <- which(grepl(measure_clean, combined_results$Measure, ignore.case = TRUE))
  }
  
  if (length(row_idx) > 0) {
    p_val_t_test <- as.character(combined_results$T_test_p_fmt[row_idx])
    p_val_wilcoxon <- as.character(combined_results$Wilcoxon_p_fmt[row_idx])
    p_val_mixed <- as.character(combined_results$Mixed_p_fmt[row_idx])
    p_val_bootstrap <- as.character(combined_results$Bootstrap_p_fmt[row_idx])
    p_val_permutation <- as.character(combined_results$Permutation_p_fmt[row_idx])
  } else {
    p_val_t_test <- p_val_wilcoxon <- p_val_mixed <- p_val_bootstrap <- p_val_permutation <- "NA"
  }
  
  # Format p-values for display
  sig_text_t_test <- paste("t-test:", p_val_t_test)
  sig_text_wilcoxon <- paste("Wilcoxon:", p_val_wilcoxon)
  sig_text_mixed <- paste("Mixed model:", p_val_mixed)
  sig_text_bootstrap <- paste("Bootstrap:", p_val_bootstrap)
  sig_text_permutation <- paste("Permutation:", p_val_permutation)
  
  # Create subtitle with multiple p-values
  subtitle <- paste0(
    sig_text_t_test, "\n",
    sig_text_wilcoxon, "\n",
    sig_text_mixed, "\n",
    sig_text_bootstrap, "\n",
    sig_text_permutation
  )
  
  # Set colors based on region
  if (region == "M") {
    fill_colors <- c("WT" = "darkblue", "KO" = "white")
    border_color <- "darkblue"
  } else {
    fill_colors <- c("WT" = "skyblue", "KO" = "white")
    border_color <- "skyblue"
  }
  
  # Create boxplot
  p <- ggplot(region_data, aes_string(x = "Genotype", y = measure, fill = "Genotype")) +
    geom_boxplot(alpha = 0.5, outlier.shape = NA, color = border_color) +
    geom_jitter(width = 0.2, alpha = 0.7, height = 0, color = border_color) +
    scale_fill_manual(values = fill_colors) +
    labs(title = paste(measure_clean, "in", ifelse(region == "M", "MD-M", "MD-L"), "Region"),
         subtitle = subtitle,
         y = measure_clean,
         x = "Genotype") +
    theme_classic(base_size = 12) +
    theme(legend.position = "none",
          plot.subtitle = element_text(size = 10))
  
  return(p)
}

# Create plots for each measure in MD-M region
mdm_plots <- lapply(measures, function(m) {
  tryCatch({
    create_plots(morphology_data, m, "M")
  }, error = function(e) {
    message("Error creating plot for measure ", m, " in MD-M region: ", e$message)
    NULL
  })
})
names(mdm_plots) <- measures

# Create plots for each measure in MD-L region
mdl_plots <- lapply(measures, function(m) {
  tryCatch({
    create_plots(morphology_data, m, "L")
  }, error = function(e) {
    message("Error creating plot for measure ", m, " in MD-L region: ", e$message)
    NULL
  })
})
names(mdl_plots) <- measures

# Remove any NULL elements (failed plots)
mdm_plots <- mdm_plots[!sapply(mdm_plots, is.null)]
mdl_plots <- mdl_plots[!sapply(mdl_plots, is.null)]

# Arrange plots in a grid
library(gridExtra)

# Check if we have plots to display
if (length(mdm_plots) > 0) {
  # Create grid of plots for MD-M region
  grid.arrange(
    grobs = mdm_plots,
    ncol = 2,
    top = "MD-M Region: Effects of Fmr1 Knockout on Dendritic Morphology (Multiple Test Approaches)"
  )
}
```

```
# Check if we have plots to display
if (length(mdl_plots) > 0) {
  # Create grid of plots for MD-L region
  grid.arrange(
    grobs = mdl_plots,
    ncol = 2,
    top = "MD-L Region: Effects of Fmr1 Knockout on Dendritic Morphology (Multiple Test Approaches)"
  )
}
```

## Summary of Findings

```
# Create a nicely formatted summary of findings
cat("\n\n========== SUMMARY OF FINDINGS ==========\n")
```

```
## 
## 
## ========== SUMMARY OF FINDINGS ==========
```

```
# For MD-M region, report measures that are significant across different tests
cat("\nMeasures in MD-M region showing significant differences between WT and KO:\n")
```

```
## 
## Measures in MD-M region showing significant differences between WT and KO:
```

```
if (exists("mdm_combined_fmt_global")) {
  for (i in 1:nrow(mdm_combined_fmt_global)) {
    measure <- mdm_combined_fmt_global$Measure[i]
    t_sig <- mdm_combined_fmt_global$T_test_p_sig[i]
    wilcox_sig <- mdm_combined_fmt_global$Wilcoxon_p_sig[i]
    mixed_sig <- mdm_combined_fmt_global$Mixed_p_sig[i]
    bootstrap_sig <- mdm_combined_fmt_global$Bootstrap_p_sig[i]
    perm_sig <- mdm_combined_fmt_global$Permutation_p_sig[i]
    
    # Handle potential NA in Cohens_d_fmt
    cohens_d_str <- mdm_combined_fmt_global$Cohens_d_fmt[i]
    cohens_d <- if(is.na(cohens_d_str) || cohens_d_str == "NA") 0 else as.numeric(cohens_d_str)
    
    # Count significant tests (p < 0.05)
    sig_count <- sum(c(t_sig, wilcox_sig, mixed_sig, bootstrap_sig, perm_sig) %in% c("*", "**", "***"), na.rm = TRUE)
    
    if (sig_count > 0) {
      effect_size_text <- case_when(
        abs(cohens_d) < 0.2 ~ "negligible",
        abs(cohens_d) < 0.5 ~ "small",
        abs(cohens_d) < 0.8 ~ "medium",
        abs(cohens_d) >= 0.8 ~ "large"
      )
      
      sig_tests <- c()
      if (t_sig %in% c("*", "**", "***")) sig_tests <- c(sig_tests, "t-test")
      if (wilcox_sig %in% c("*", "**", "***")) sig_tests <- c(sig_tests, "Wilcoxon")
      if (mixed_sig %in% c("*", "**", "***")) sig_tests <- c(sig_tests, "Mixed model")
      if (bootstrap_sig %in% c("*", "**", "***")) sig_tests <- c(sig_tests, "Bootstrap")
      if (perm_sig %in% c("*", "**", "***")) sig_tests <- c(sig_tests, "Permutation")
      
      cat(sprintf("- %s: Significant in %d/%d tests (%s) with Cohen's d = %.2f (%s effect)\n", 
                 measure, 
                 sig_count, 5,
                 paste(sig_tests, collapse = ", "),
                 cohens_d,
                 effect_size_text))
    }
  }
  
  # If no measures are significant
  all_sigs <- c(mdm_combined_fmt_global$T_test_p_sig, 
               mdm_combined_fmt_global$Wilcoxon_p_sig, 
               mdm_combined_fmt_global$Mixed_p_sig,
               mdm_combined_fmt_global$Bootstrap_p_sig,
               mdm_combined_fmt_global$Permutation_p_sig)
  
  if (sum(all_sigs %in% c("*", "**", "***"), na.rm = TRUE) == 0) {
    cat("No measures showed significant differences at p < 0.05 in any test.\n")
  }
} else {
  cat("No combined results available for MD-M region.\n")
}
```

```
## - Terminals: Significant in 5/5 tests (t-test, Wilcoxon, Mixed model, Bootstrap, Permutation) with Cohen's d = 0.81 (large effect)
## - NodesCount: Significant in 5/5 tests (t-test, Wilcoxon, Mixed model, Bootstrap, Permutation) with Cohen's d = 0.76 (medium effect)
## - Total Dendritic Length (Μm): Significant in 5/5 tests (t-test, Wilcoxon, Mixed model, Bootstrap, Permutation) with Cohen's d = 0.75 (medium effect)
## - DendriticComplexity: Significant in 5/5 tests (t-test, Wilcoxon, Mixed model, Bootstrap, Permutation) with Cohen's d = 0.63 (medium effect)
```

```
# For MD-L region, report measures that are significant across different tests
cat("\nMeasures in MD-L region showing significant differences between WT and KO:\n")
```

```
## 
## Measures in MD-L region showing significant differences between WT and KO:
```

```
if (exists("mdl_combined_fmt_global")) {
  for (i in 1:nrow(mdl_combined_fmt_global)) {
    measure <- mdl_combined_fmt_global$Measure[i]
    t_sig <- mdl_combined_fmt_global$T_test_p_sig[i]
    wilcox_sig <- mdl_combined_fmt_global$Wilcoxon_p_sig[i]
    mixed_sig <- mdl_combined_fmt_global$Mixed_p_sig[i]
    bootstrap_sig <- mdl_combined_fmt_global$Bootstrap_p_sig[i]
    perm_sig <- mdl_combined_fmt_global$Permutation_p_sig[i]
    
    # Handle potential NA in Cohens_d_fmt
    cohens_d_str <- mdl_combined_fmt_global$Cohens_d_fmt[i]
    cohens_d <- if(is.na(cohens_d_str) || cohens_d_str == "NA") 0 else as.numeric(cohens_d_str)
    
    # Count significant tests (p < 0.05)
    sig_count <- sum(c(t_sig, wilcox_sig, mixed_sig, bootstrap_sig, perm_sig) %in% c("*", "**", "***"), na.rm = TRUE)
    
    if (sig_count > 0) {
      effect_size_text <- case_when(
        abs(cohens_d) < 0.2 ~ "negligible",
        abs(cohens_d) < 0.5 ~ "small",
        abs(cohens_d) < 0.8 ~ "medium",
        abs(cohens_d) >= 0.8 ~ "large"
      )
      
      sig_tests <- c()
      if (t_sig %in% c("*", "**", "***")) sig_tests <- c(sig_tests, "t-test")
      if (wilcox_sig %in% c("*", "**", "***")) sig_tests <- c(sig_tests, "Wilcoxon")
      if (mixed_sig %in% c("*", "**", "***")) sig_tests <- c(sig_tests, "Mixed model")
      if (bootstrap_sig %in% c("*", "**", "***")) sig_tests <- c(sig_tests, "Bootstrap")
      if (perm_sig %in% c("*", "**", "***")) sig_tests <- c(sig_tests, "Permutation")
      
      cat(sprintf("- %s: Significant in %d/%d tests (%s) with Cohen's d = %.2f (%s effect)\n", 
                 measure, 
                 sig_count, 5,
                 paste(sig_tests, collapse = ", "),
                 cohens_d,
                 effect_size_text))
    }
  }
  
  # If no measures are significant
  all_sigs <- c(mdl_combined_fmt_global$T_test_p_sig, 
               mdl_combined_fmt_global$Wilcoxon_p_sig, 
               mdl_combined_fmt_global$Mixed_p_sig,
               mdl_combined_fmt_global$Bootstrap_p_sig,
               mdl_combined_fmt_global$Permutation_p_sig)
  
  if (sum(all_sigs %in% c("*", "**", "***"), na.rm = TRUE) == 0) {
    cat("No measures showed significant differences at p < 0.05 in any test.\n")
  }
} else {
  cat("No combined results available for MD-L region.\n")
}
```

```
## No measures showed significant differences at p < 0.05 in any test.
```

```
# Compare effect sizes between MD-M and MD-L regions
cat("\nComparison of effect sizes (Cohen's d) between MD-M and MD-L regions:\n")
```

```
## 
## Comparison of effect sizes (Cohen's d) between MD-M and MD-L regions:
```

```
if (exists("mdm_combined_fmt_global") && exists("mdl_combined_fmt_global")) {
  # Get formatted measure names from the original measures
  formatted_measures <- sapply(measures, function(m) {
    measure_name <- gsub("_", " ", m)
    measure_name <- gsub("in um", "(µm)", measure_name)
    measure_name <- tools::toTitleCase(measure_name)
    return(measure_name)
  })
  
  for (measure_name in formatted_measures) {
    mdm_idx <- which(mdm_combined_fmt_global$Measure == measure_name)
    mdl_idx <- which(mdl_combined_fmt_global$Measure == measure_name)
    
    if (length(mdm_idx) > 0 && length(mdl_idx) > 0) {
      mdm_d_str <- mdm_combined_fmt_global$Cohens_d_fmt[mdm_idx]
      mdl_d_str <- mdl_combined_fmt_global$Cohens_d_fmt[mdl_idx]
      
      # Handle potential NA in Cohens_d_fmt
      mdm_d <- if(is.na(mdm_d_str) || mdm_d_str == "NA") 0 else as.numeric(mdm_d_str)
      mdl_d <- if(is.na(mdl_d_str) || mdl_d_str == "NA") 0 else as.numeric(mdl_d_str)
      
      mdm_effect <- case_when(
        abs(mdm_d) < 0.2 ~ "negligible",
        abs(mdm_d) < 0.5 ~ "small",
        abs(mdm_d) < 0.8 ~ "medium",
        abs(mdm_d) >= 0.8 ~ "large"
      )
      
      mdl_effect <- case_when(
        abs(mdl_d) < 0.2 ~ "negligible",
        abs(mdl_d) < 0.5 ~ "small",
        abs(mdl_d) < 0.8 ~ "medium",
        abs(mdl_d) >= 0.8 ~ "large"
      )
      
      cat(sprintf("- %s: MD-M = %.2f (%s effect), MD-L = %.2f (%s effect)\n", 
                 measure_name, mdm_d, mdm_effect, mdl_d, mdl_effect))
    } else {
      cat(sprintf("- %s: Data not available for one or both regions\n", measure_name))
    }
  }
} else {
  cat("Effect size comparison not available - missing combined results.\n")
}
```

```
## - Total Dendritic Length (Μm): MD-M = 0.75 (medium effect), MD-L = -0.06 (negligible effect)
## - DendriticComplexity: MD-M = 0.63 (medium effect), MD-L = 0.02 (negligible effect)
## - Number of Primary Dendrites: MD-M = 0.27 (small effect), MD-L = -0.24 (small effect)
## - NodesCount: MD-M = 0.76 (medium effect), MD-L = -0.11 (negligible effect)
## - Terminals: MD-M = 0.81 (large effect), MD-L = -0.15 (negligible effect)
```
